# Photodissociation Spectroscopy and Photofragment Imaging to Probe Fe^+^(Benzene)_1,2_ Dissociation Energies

**DOI:** 10.1021/acs.jpca.3c00735

**Published:** 2023-03-15

**Authors:** Jason E. Colley, Nathan J. Dynak, John R. C. Blais, Michael A. Duncan

**Affiliations:** Department of Chemistry, University of Georgia, Athens, Georgia 30602, United States

## Abstract

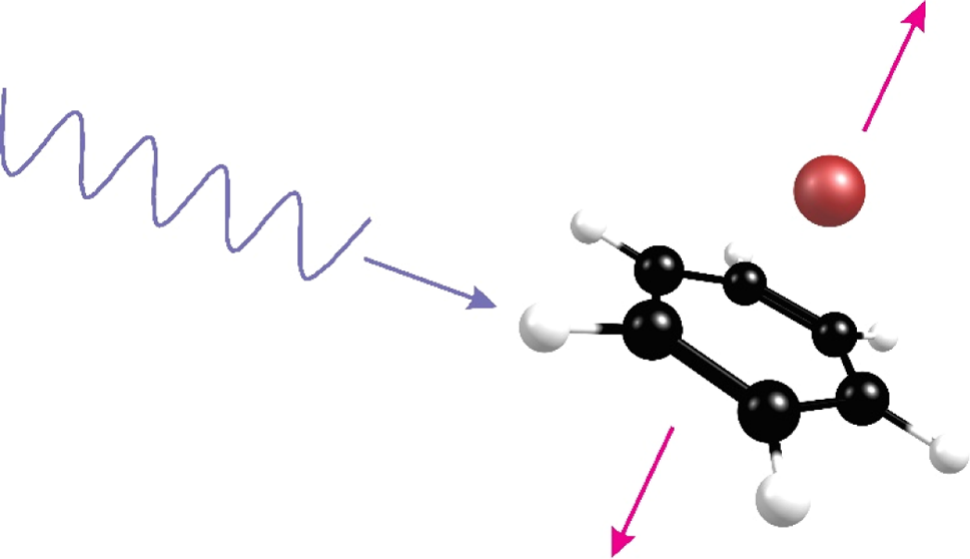

Tunable laser photodissociation spectroscopy measurements and photofragment imaging experiments are employed to investigate the dissociation energy of the Fe^+^(benzene) ion–molecule complex. Additional spectroscopy measurements determine the dissociation energy of Fe^+^(benzene)_2_. The dissociation energies for Fe^+^(benzene) determined from the threshold for the appearance of the Fe^+^ fragment (48.4 ± 0.2 kcal/mol) and photofragment imaging (≤49.3 ± 3.2 kcal/mol) agree nicely with each other and with the value determined previously by collision-induced dissociation (49.5 ± 2.9 kcal/mol), but they are lower than the values produced by computational chemistry at the density functional theory level using different functionals recommended for transition-metal chemistry. The threshold measurement for Fe^+^(benzene)_2_ (43.0 ± 0.2 kcal/mol) likewise agrees with the value (44.7 ± 3.8 kcal/mol) from previous collision-induced dissociation measurements.

## Introduction

Transition metal–benzene complexes and their corresponding sandwiches provide classic examples of organometallic bonding.^1−8^ The intrinsic bonding properties of these systems are often studied in the absence of solvents in the form of gas phase ions. Mass spectrometry has investigated the reactions, thermochemistry, and photochemistry of these species, whereas spectroscopy and computational chemistry have investigated their electronic structure and bonding.^9−59^ The metal–ligand bond thermochemistry of these complexes is fundamental to their understanding but problematic for both experiments and theory. Experimental measurements most often employ collision-induced dissociation (CID),^16,28,35^ which is subject to appropriate modeling of the collisional dissociation rate as a function of energy. Computational chemistry typically employs density functional theory (DFT) with basis sets and functionals optimized for transition metals.^14,21,22,27,29,30,36−41,50,54−60^ In the present report, we employ two different experimental approaches to investigate the dissociation behavior of Fe^+^(benzene)_*n*_ complexes. Tunable laser threshold spectroscopy and photofragment imaging provide new measurements of dissociation energies for comparison with previous experiments and the predictions of theory.

Metal ion–benzene complexes have been studied with a variety of experimental methods to determine their dissociation energies, and data are available for many M^+^(benzene)_1,2_ complexes.^16,28,32,34,35^ The most common method is CID, in which the energy dependence of collisional fragmentation is measured, and the rate of dissociation versus the energy is modeled using statistical rate theory.^16,28,35^ The dissociation usually becomes detectable only at energies well above the actual threshold for bond breaking. This so-called “kinetic shift” can be accounted for using assumptions about the energy-transfer efficiency in collisions, the unimolecular dissociation rate, and the threshold energy for dissociation. Agreement between the measured dissociation yield as a function of energy and that predicted by the modeling provides the dissociation energy. In many cases, this strategy for determining bond energies has been successful, but it becomes less reliable for larger molecules with greater kinetic shifts on their thresholds. Metal ion–benzene complexes are large enough that statistical modeling is potentially problematic, and it is desirable to have bond energies measured using methods other than CID.

Experiments and theory on transition-metal ion–molecule complexes are complicated by the many low-lying electronic states and different spin states. Iron and its complexes are particularly challenging in this regard. The isolated metal ion has a 3d^6^4s^1^ (^6^D) ground state, with nearby 3d^7^ (^4^F), 3d^6^4s^1^ (^4^D), and 3d^7^ (^4^P) excited states.^61^ The ground state of the Fe^+^(benzene) complex is predicted by theory to have *C*_2*v*_ symmetry and to be a ^4^A_1_ state,^27,36,37^ correlating to the ground-state benzene and the excited-state ^4^F atomic iron cation. It is therefore not clear how collisional dissociation takes place (i.e., on which potential energy surface) and how its threshold should be interpreted. The density of low-lying excited states introduces severe multireference character into computations, also making it difficult to obtain reliable values for dissociation energies from theory. This system has therefore been the subject of several computational studies employing different computational approaches.^27,36,37^

In the present study, we investigate the dissociation energies of Fe^+^(benzene) and Fe^+^(benzene)_2_ employing different experimental methods. We use tunable laser photodissociation spectroscopy in the visible wavelength region to investigate both of these ions. If there is a sufficient density of excited electronic states with coupling to the dissociation coordinate, the first energy at which dissociation occurs provides a direct measure of the dissociation threshold, i.e., the bond energy. If these conditions do not apply, the method provides an upper limit to the bond energy. This method of determining the dissociation energy has been applied previously to transition-metal complexes and to their metal dimers.^62−68^ In a second method applicable only to Fe^+^(benzene), we employ photofragment imaging of the benzene cation produced at higher-energy excitation via a charge-transfer dissociation process. We have described this method previously in studies of silver ion complexes with benzene and other similar aromatic ligands.^59,60^ These methods provide determinations of the dissociation energy for these complexes independent from previous CID measurements. To complement these experiments, we have conducted computational studies with DFT and several functionals designed and optimized for transition-metal chemistry.

## Methods

Ion–molecule complexes of the form Fe^+^(benzene)_*n*_ were produced by laser vaporization^69^ in a pulsed supersonic expansion of argon containing benzene vapor at its ambient concentration above the room-temperature liquid. Ions produced in this way are typically believed to have rotational temperatures of 10–50 K.^69^ The ions were analyzed and mass selected for study with a reflectron time-of-flight mass spectrometer designed for photodissociation experiments.^70,71^ Mass selection was accomplished with pulsed deflection plates in the first flight tube of the reflectron instrument, photodissociation took place at the turning point in the reflectron field, and fragment mass analysis was accomplished using the flight time through a second drift-tube section. Tunable UV–visible radiation for threshold spectroscopy experiments was provided by a Nd:YAG-pumped optical parametric oscillator (OPO) laser system (Continuum Horizon II; line width 5–7 cm^–1^; 1.0 mJ/pulse energy; unfocused). The yield of the fragment mass recorded versus the photon energy provided the photodissociation spectrum. The laser step size for survey scans was 1 nm, whereas that for scans of the threshold regions was 0.1 nm.

Photofragment imaging studies were conducted using our selected-ion velocity-map imaging (SI-VMI) instrument.^59,60^ In this device, ions are selected by their flight time though a linear time-of-flight instrument and then transmitted into an imaging flight tube where photodissociation occurs. The photodissociation laser was a Nd:YAG (Spectra-Physics GCR-170) operating on the fourth harmonic wavelength (266 nm; 4.66 eV; 5 mJ/pulse; unfocused). Photofragment ions were reaccelerated using a series of electrostatic lenses designed for VMI^72−77^ and detected using the DC-slice imaging method.^78^ To achieve slicing, the dual MCP/P-47 phosphor detector (Beam Imaging Solutions BOS-75) was activated in a narrow time window with a fast rise-time high-voltage pulser (DEI PVX-4140), allowing fragment ions in the central ∼90 ns of the arrival-time distribution to be detected. Images were collected using a CCD camera (Edmund Optics), averaging over several hundred thousand laser shots. Images were processed with the NuACQ and BasisFit software.^79^ Calibration was accomplished by measuring the image of Ar^+^ from the photodissociation of Ar_2_^+^ using the same instrument settings.^80^ The design for this instrument using photofragment imaging of jet-cooled ions that are mass-selected is unique to our lab,^59,60^ but similar instruments have recently been reported by other groups.^81−87^

Computational studies on the iron cation–benzene complexes were carried out with the Gaussian 16 program package,^87^ using DFT with the def2-TZVP basis set.^89^ Calculations were performed using several different functionals (B3LYP, M06-L,^90,91^ and MN15-L^92^). All energetics, i.e., dissociation energies, were zero-point-corrected.

## Results and Discussion

Laser vaporization produces a distribution of cation–molecular complexes of the form Fe^+^(benzene)_*n*_. A typical mass spectrum is presented as Figure S1 in the Supporting Information. Photodissociation of Fe^+^(benzene) in the visible wavelength region produces only the Fe^+^ photofragment. At higher energies in the UV, the benzene cation is also observed as a photofragment, occurring via a charge-transfer process. These fragmentation channels, which were reported in previous work,^13^ are shown in Figures S2 and S3 in the Supporting Information. Photodissociation of the Fe^+^(benzene)_2_ complex produces the Fe^+^(benzene) fragment via the elimination of benzene and a small amount of the Fe^+^ fragment, presumably through a sequential elimination of benzene from Fe^+^(benzene). This is shown in Figure S4 in the Supporting Information.

### Photodissociation Spectroscopy

**Figure 1:**
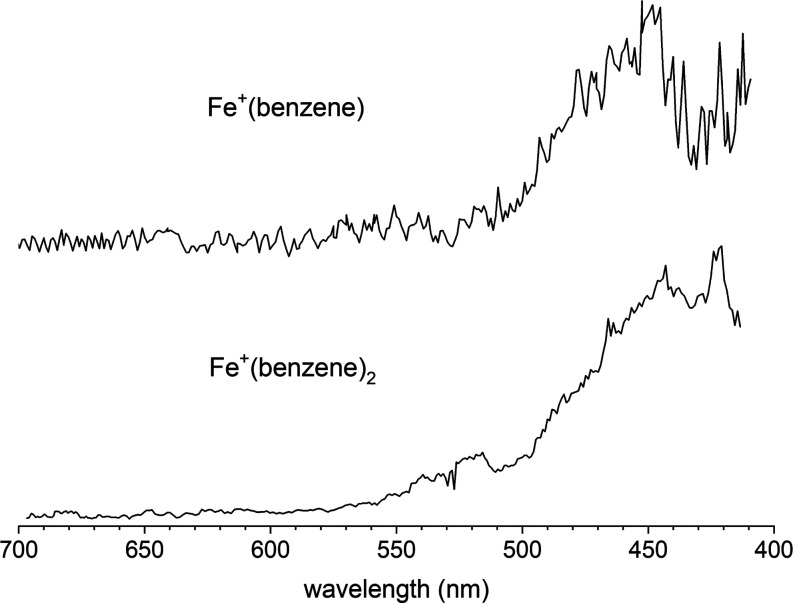
Photodissociation spectra in the visible wavelength region of Fe^+^(benzene) (top) and Fe^+^(benzene)_2_ (bottom), measured in the Fe^+^ and Fe^+^(benzene) fragment ion channels, respectively. The laser step size was 1 nm.

Figure 1 shows the photodissociation spectra of the Fe^+^(benzene) and Fe^+^(benzene)_2_ ions in the 700–400 nm region. The Fe^+^(benzene) spectrum was recorded in the Fe^+^ fragment ion mass channel, whereas the Fe^+^(benzene)_2_ spectrum was recorded in the Fe^+^(benzene) fragment ion mass channel. The respective fragment ions are not produced at levels above the background at lower energies but exhibit an onset in the visible wavelength region, after which there is essentially continuous fragmentation and greater signal intensity toward higher energy. The intensity increases significantly for both ions at wavelengths shorter than 500 nm. The production of the Fe^+^ fragment ion from Fe^+^(benzene) continues and becomes more intense in the UV up to at least 220 nm (Figure S5, Supporting Information). Additionally, a benzene cation photofragment is also detected from this same parent ion in the 280–240 nm region (Figure S6, Supporting Information).

To investigate the source of these absorption/fragmentation signals, we have performed computational studies with DFT on the electronic structures and bonding configurations of these ions. The energetics from these computational studies are presented in Table 1, including the results from three DFT functionals on the doublet, quartet, and sextet spin states for each complex. Consistent with previous work, all three functionals agree that the quartet is the ground state for Fe^+^(benzene). B3LYP and MN15-L functionals also found that the quartet is the ground state for Fe^+^(benzene)_2_, whereas the M06-L functional found the doublet. Also, consistent with previous work using DFT, we found that the ground state of Fe^+^(benzene) is distorted slightly into *C*_2*v*_ symmetry with an ^4^A_1_ configuration. However, multireference computations by Lanza et al. have previously found that the ground state has an ^4^A_2_ symmetry.^58^ Additional details of the computations are presented in the Supporting Information.

**Table 1:** Computed Energetics for Fe^+^(Benzene)_1,2_ Complexes Employing Different DFT Functionals^a^

energies	B3LYP	M06-L	MN15-L
benzene	–232.237193	–232.193605	–232.022192
Fe^+^ (*m* = 2)	–1263.369688	–1263.283754	–1263.228030
Fe^+^ (*m* = 4)	–1263.411346	–1263.323876	–1263.304679
Fe^+^ (*m* = 6)	–1263.404095	–1263.310728	–1263.311894
Fe^+^(benzene) (*m* = 2)	–1495.702108	–1495.604750	–1495.394283
Fe^+^(benzene) (*m* = 4)	–1495.735843	–1495.628604	–1495.443364
Fe^+^(benzene) (*m* = 6)	–1495.703477	–1495.587757	–1495.422972
Fe^+^-(benzene)_2_ (*m* = 2)	–1728.010477	–1727.898373	–1727.534614
Fe^+^-(benzene)_2_ (*m* = 4)	–1728.023016	–1727.894086	–1727.550259
Fe^+^-(benzene)_2_ (*m* = 6)	–1727.956849	–1727.820005	–1727.477227

dissociation energies	B3LYP	M06-L	MN15-L
Fe^+^-(benzene) (*m* = 2)	59.75	79.94	90.40
Fe^+^-(benzene) (*m* = 4)	54.78	69.73	73.10
Fe^+^-(benzene) (*m* = 6)	39.02	52.35	55.78
Fe^+^-(benzene)_2_ (*m* = 2)	44.66	62.76	74.13
Fe^+^-(benzene)_2_ (*m* = 4)	31.36	45.10	53.15
Fe^+^-(benzene)_2_ (*m* = 6)	10.15	24.35	20.12

a Energies (zero-point-corrected) are in hartree, except for dissociation energies which are in kcal/mol. The dissociation energies are for the potential energy surface corresponding to the indicated spin state. Corrections are required for the respective atomic excited-state energies to derive the adiabatic values. *m* = 2*s* + 1 indicates the multiplicity.

**Figure 2:**
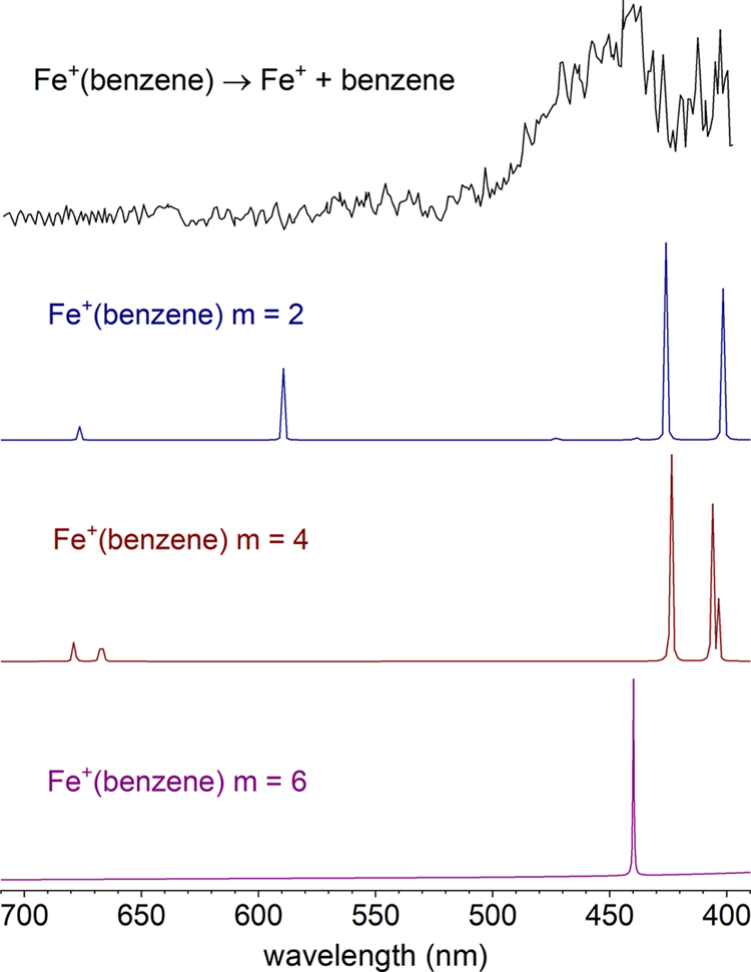
Photodissociation spectrum of Fe^+^(benzene) (top) compared to the electronic absorption spectra predicted by TD-DFT using the B3LYP functional for doublet, quartet, and sextet spin states. The line widths in the predicted spectra are for visual appeal and have no specific significance.

Time-dependent DFT (TD-DFT) computations using the B3LYP functional were conducted on the electronic spectroscopy for both of these ions. Electronic spectra were predicted for the doublet, quartet and sextet spin states. Figure 2 shows the comparison of the measured photodissociation spectrum with the absorption spectra predicted by TD-DFT for the Fe^+^(benzene) ion. As indicated, both the doublet and quartet species have several transitions predicted at low energy, whereas the sextet species has only one. The experimental spectrum does not look like any of the predicted spectra. This is partly because the spectra predicted by theory only include electronic band origins and do not include any vibronic structure that would be expected with such electronic transitions. It is also true that weaker transitions are predicted that are not evident in these spectra because of their low relative intensities (see the Supporting Information). Both the doublet and quartet species have much stronger transitions predicted in the 450–400 nm region where the experiment has more intense signal. However, beyond this point, there is not enough information from our TD-DFT theory to develop any preference for a spin state that agrees with the experiment.

Multireference wave function studies of the electronic spectroscopy of Fe^+^(benzene) were conducted previously by Lanza et al. to identify any possible coincidences between Fe^+^(benzene) transitions and visible diffuse interstellar bands (DIBs).^58^ They found seven relatively more intense electronic transitions in the 800–400 nm region, with many other weaker transitions in this same region, producing an almost continuous density of states, although the oscillator strengths of all of the transitions were low. This is more consistent with the continuous spectrum that we measure than the sparse distribution of transitions predicted by TD-DFT. Although Lanza et al. found close coincidences between their predicted spectrum and some DIBs, the continuous experimental spectrum that we measure effectively rules out any assignment to DIBs for the Fe^+^(benzene) ion. Similar multireference studies are not available for Fe^+^(benzene)_2_, but we can speculate that it has a density of electronic states somewhat similar to that of Fe^+^(benzene), explaining its continuous spectrum.

**Figure 3:**
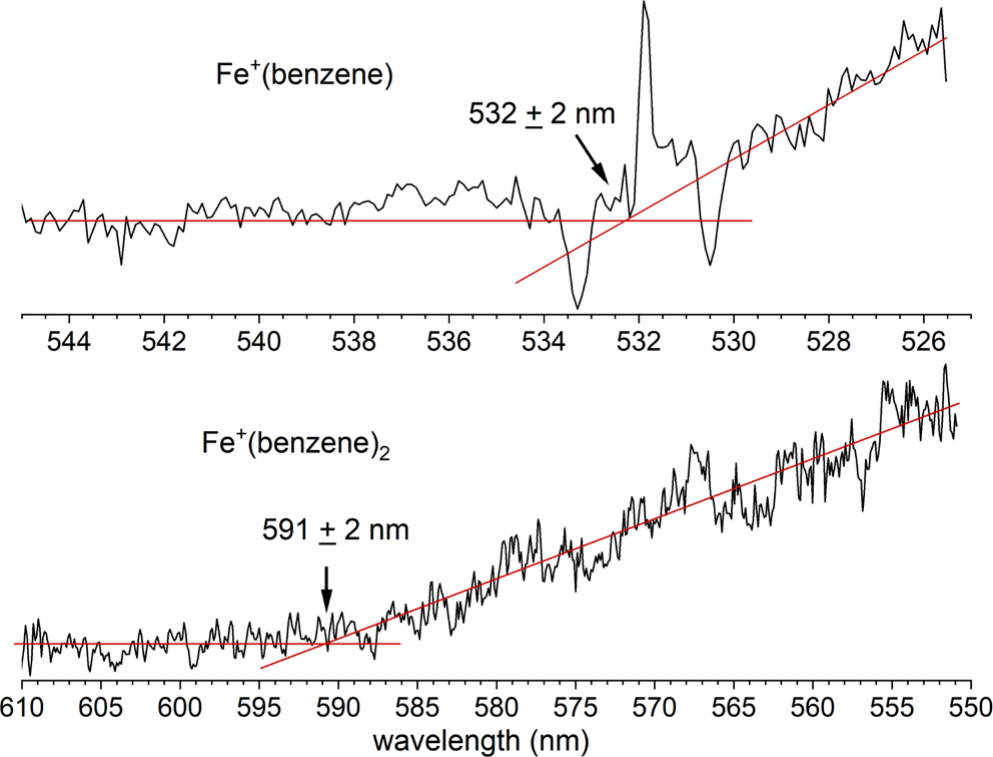
Photodissociation spectra of Fe^+^(benzene) (top) and Fe^+^(benzene)_2_ (bottom) in the respective threshold wavelength regions. The laser step size was 0.1 nm.

Both of the Fe^+^(benzene) and Fe^+^(benzene)_2_ ions exhibit an initial onset in their photodissociation signals at the low energy end of these spectra. Figure 3 shows an expanded view of the threshold region for these two ions, scanned with a smaller step size (0.1 nm) than that used with the broader scans (1 nm). To determine the thresholds, we use an averaged signal level line for the baseline prior to the onset and a second averaged line for the rising signal, and set the threshold at the intersection of these lines. There is no theoretical basis for such a linear threshold dependence; we just use this to guide the eye to find the sudden change in the signal level to determine the position of the threshold. The data for Fe^+^(benzene) is affected by the sudden change in the OPO signal near 532 nm, where the tuning crystal has a degeneracy point and the power level correction is difficult to obtain. This accounts for the dips in intensity near 533.3 and 530.5 nm and the peak at 531.7 nm. The data at the threshold for Fe^+^(benzene)_2_ is more well-behaved. In both cases though, the thresholds can be determined with the baseline/signal line intersection method. The fragmentation threshold for Fe^+^(benzene) is found to be 532 ± 2 nm, whereas that for Fe^+^(benzene)_2_ is 591 ± 2 nm. The error bars in these values are estimated from the noise level in the spectra in the threshold region; the laser line width is much smaller than this. Because photodissociation occurs at these threshold wavelengths, the dissociation energies of these ions must be less than or equal to the corresponding energies. Strictly speaking, both thresholds therefore represent upper limits on the dissociation energies. However, if there is a sufficient density of vibronic states in the threshold region, which is suggested to be true by Lanza et al.’s theory on Fe^+^(benzene), and if there is efficient coupling for the levels which absorb to dissociative levels, these thresholds may correspond to the true thermodynamic dissociation energy values. Consistent with this, there are no gaps or structure in the dissociation spectra at energies above the thresholds.

**Figure 4:**
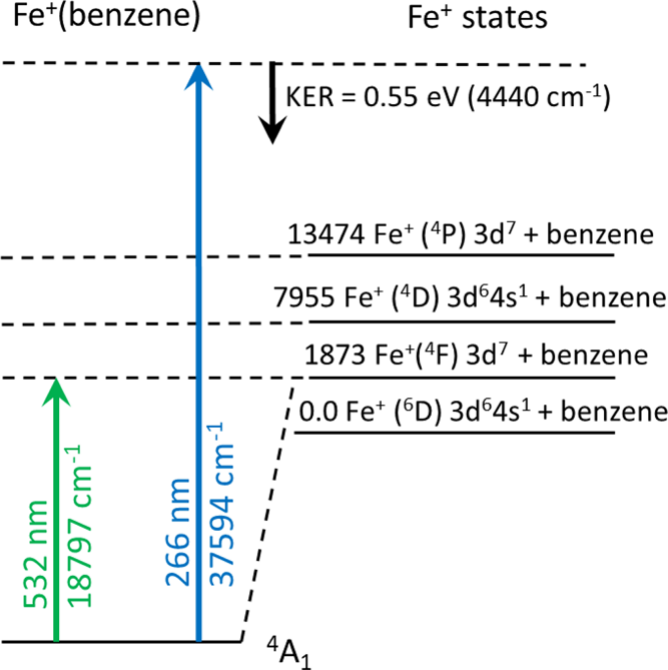
Schematic level diagram for the energy levels of Fe^+^(benzene) compared to those of the Fe^+^ atomic ion. The green arrow at the left shows the dissociation threshold measured in the tunable laser experiment. The right blue arrow shows the excitation energy used for the photofragment imaging experiment. The black downward arrow shows the maximum kinetic energy measured for the Fe^+^ photofragment.

The threshold wavelengths correspond to upper limits of the dissociation energies of 53.7 and 48.4 kcal/mol, respectively, for Fe^+^(benzene) and Fe^+^(benzene)_2_. However, it is important to note that the ground states of both of these ions are quartets and therefore optical transitions are only allowed to excited states which are also quartets. The thresholds determined by the scanned threshold method therefore represent the dissociation energies for each of these ions on their quartet potential energy surfaces, which correlate with the ^4^F atomic state of Fe^+^, as shown in the left side of the level diagram in Figure 4. To obtain the adiabatic dissociation energies, i.e., the energy with respect to Fe^+^ in its ground ^6^D state and benzene in its ground state, we must subtract off the energy of the Fe^+^ (^4^F – ^6^D) interval (1873 cm^–1^). With this adjustment, the adiabatic dissociation energy upper limit for Fe^+^(benzene) is 48.4 ± 0.2 kcal/mol and that for Fe^+^(benzene)_2_ is 43.0 ± 0.2 kcal/mol. It is conceivable that more complex dynamics occurs at higher energies, where excitation may lead to direct production of ground-state Fe^+^ and benzene, perhaps with an associated barrier. However, there is no evidence in our data for this. The simplest process likely to produce the signal at the threshold is dissociation on the quartet surface, as described here.

These dissociation energies can be compared to values obtained previously for these ions using other methods and to the results of computational chemistry. Table 2 presents this comparison. As is shown, the threshold energies determined here agree quite well with dissociation energies determined previously. Our values for both Fe^+^(benzene) and Fe^+^(benzene)_2_ are within the error bars of the CID values determined by Meyer, Khan, and Armentrout.^16^ Our value for Fe^+^(benzene) is quite close to our computed dissociation energy using the B3LYP functional but much less than the values determined with other functionals. The threshold value for Fe^+^(benzene)_2_ agrees reasonably well with the previous experimental value (44.7 ± 3.8 kcal/mol) but does not agree with the computed dissociation energies, which are quite variable with different functionals.

**Table 2:** Dissociation Energies for Fe^+^(Benzene) and Fe^+^(Benzene)_2_ Determined from Different Experiments and from Theory^a^

complex	scanned threshold	CT image	CID	theory
Fe^+^(benzene)	48.4 ± 0.2	49.3 ± 3.2	49.5 ± 2.9^28^	51.1^14^
				49.2^27^
				56.4^36^
				49.0^37^
				49.4^b^
Fe^+^(benzene)_2_	43.0 ± 0.2		44.7 ± 3.8^28^	40.6^37^
				31.4^b^

a The values given are adiabatic, for dissociation of the form Fe^+^(benzene) → Fe^+^ + benzene, or Fe^+^(benzene)_2_ → Fe^+^(benzene) + benzene, with products in their respective ground states. Energies are in kcal/mol.

b Present work, B3LYP functional.

These data provide an interesting comparison between the specific analysis protocols used here and those used with the CID experiments. To interpret the scanned threshold data, we assume that optical excitation is causing dissociation on the quartet potential energy surfaces and then we correct for the quartet–sextet atomic spacing to get the adiabatic dissociation energies. We assume that dissociation is prompt (i.e., no unimolecular kinetic time delay) because the excited states are strongly coupled and any absorption leads directly to dissociation. In the CID experiment, the dissociation energy is derived from the kinetic analysis of the threshold, with its inherent kinetic shift. Dissociation is assumed to access all spin states equally, and there is no quartet–sextet correction used to derive the adiabatic dissociation energies. The agreement between these two experiments (and the imaging experiment described below) apparently indicates that these issues are being handled correctly in both experiments. A similar conclusion was arrived at in our recent work on the Fe^+^(acetylene) complex.^67^

### Photofragment Imaging

As noted earlier, photodissociation of Fe^+^(benzene) at low energies produces only the Fe^+^ photofragment (Supporting Information Figure S2), but at higher energies, e.g., 266 nm, the charge-transfer channel producing benzene^+^ is detected (Supporting Information Figure S3). Photofragment imaging allows the energetics of these two processes to be investigated. Photodissociation of ions like Fe^+^(benzene) is complex because of the high density of excited electronic states and their overlapping energies. It is therefore usually not possible to determine exactly which state is excited at any particular wavelength. The Fe^+^ photofragment could be produced in any number of its many possible excited states, and the benzene fragment could have internal energy. This uncertainty limits the information that can be obtained from the measurement of kinetic energy release (KER) in the Fe^+^ photofragment. A simplification occurs if there is an excitation which produces a charge-transfer process, i.e., when Fe^+^(benzene) dissociates to produce Fe + benzene^+^. In this case, the excitation energy is used to break the bond and to transfer the charge, and there is therefore much less excess energy available. Because of this, the neutral iron atom produced is likely to be in its ground electronic state or one of only a few possible excited states at low energies. If the benzene cation is produced with kinetic energy, even less energy is available for excitation of the neutral metal atom. In such a case, measurement of the KER for the benzene cation allows an analysis providing information on the Fe^+^–benzene bond energy. This kind of experiment has been described recently for several Ag^+^(aromatic) ion–molecule complexes.^59,60^

**Figure 5:**
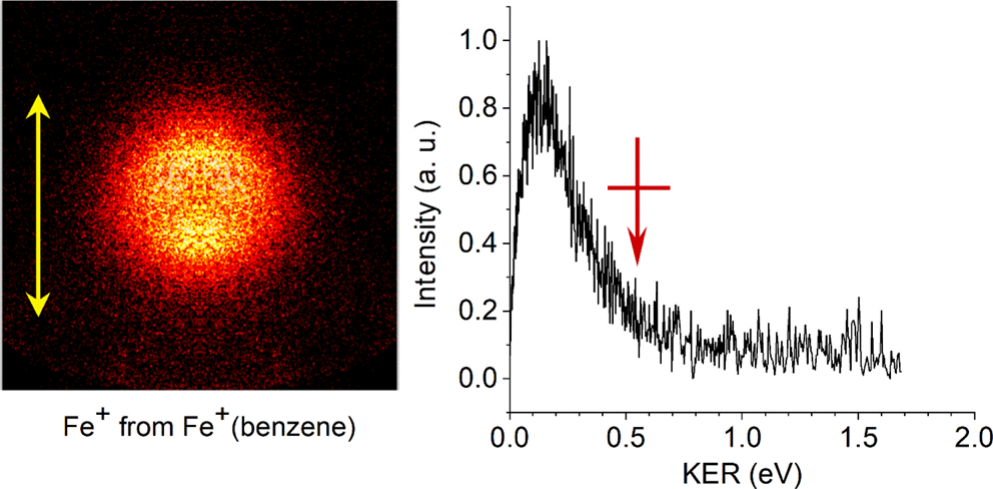
Photofragment image of the Fe^+^ fragment from the photodissociation of Fe^+^(benzene) at 266 nm (left) and the kinetic energy spectrum derived from this image (right). The vertical yellow arrow shows the dissociation laser polarization.

The left side of Figure 5 shows the image of the Fe^+^ photofragment from the photodissociation of Fe^+^(benzene) at 266 nm. This image is detected with VMI using our SI photofragment imaging instrument. The right side of the figure shows the kinetic energy spectrum derived from this image. The image is isotropic, with an angular distribution described by β = −0.08, as shown in Figure S7 in the Supporting Information. The most probable value of the kinetic energy is about 0.15 eV, whereas the maximum value is assigned to be KER_max_ = 0.55 ± 0.14 eV. The red arrow in the figure shows the assigned KER_max_ value, and the horizontal line shows the instrument resolution. The KER_max_ value is assigned by setting the upper limit of the instrument resolution width (±0.14 eV; caused by limitations in the ion optics^80^) at the point where the signal rises above the baseline, and then the KER_max_ value is set at the center of this resolution element.

The right side of Figure 4 shows how the excitation energy at 266 nm (4.66 eV; 37,594 cm^–1^) compares to the threshold energies determined from the tunable laser experiment and to the Fe^+^ electronic states. As is shown, there is enough energy to break the bond and to leave the Fe^+^ cation in any one of several possible excited states. As also shown in Figure 4, relatively little of the excess energy above that required for bond breaking appears as KER, and therefore the remainder must be present as internal (vibrational/rotational) excitation of the benzene molecule or electronic excitation of the Fe^+^. Because the Fe^+^(benzene) complex is jet cooled prior to study, electronic excitation should be favored to excited states with relatively low vibrational excitation, and therefore electronic excitation of the Fe^+^ in its ^4^P or ^4^D excited states seems likely.

**Figure 6:**
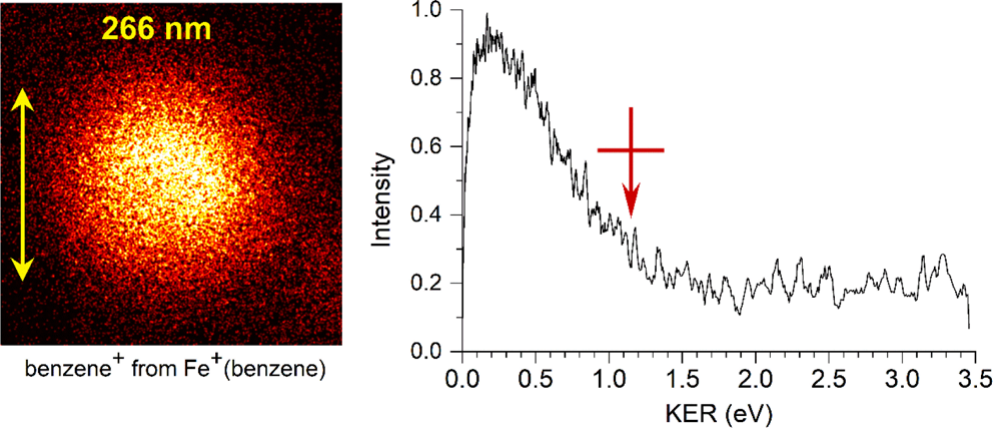
Photofragment image of the benzene^+^ fragment from the photodissociation of Fe^+^(benzene) at 266 nm (left) and the kinetic energy spectrum derived from this image (right). The vertical yellow arrow shows the dissociation laser polarization.

Figure 6 shows the image of the benzene^+^ photofragment that is also produced with photodissociation of Fe^+^(benzene) at 266 nm. This image shows that the photodissociation process has broken the bond and transferred the charge and that enough excess energy is available to eject the benzene cation from the dissociation position with kinetic energy. To produce this benzene cation photofragment, the photon energy must exceed the bond energy (*D*_0_) and the ionization potential difference between Fe (IP = 7.87 eV) and benzene (IP = 9.24 eV),^93,94^ i.e., ΔIP = 1.37 eV, and any excess energy is available for KER. The right side of the figure shows the kinetic energy spectrum derived from this image. Much of the signal occurs at a lower kinetic energy, with a most probable value of about 0.2 eV. Allowing for the instrument resolution bandwidth, as discussed previously, the maximum KER value, which corresponds to the outside edge of the image, is KER_max_ = 1.15 ± 0.14 eV. The image is isotropic, with an angular distribution described by β = −0.13, as shown in Figure S8 in the Supporting Information.

This image does not necessarily account for all the excess energy above that required for bond breaking and charge transfer; it shows that at least this much was available for KER. In reality, it is possible that some of the excess energy was produced in vibrationally excited states of the benzene molecule or in electronically excited states of the resulting neutral iron atom. Taking all of this into account, the KER_max_ value allows the determination of an upper limit on the bond energy via the relationship
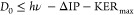
In the present system, the photon energy (4.66 eV), the ΔIP value (1.37 eV), and the KER_max_ value (1.15 eV) lead to an upper limit on the bond energy of 2.14 ± 0.14 eV, or 49.3 ± 3.2 kcal/mol. Considering the error bars, this upper limit from the charge-transfer image is essentially the same as that derived from the scanned-laser spectroscopic threshold (48.4 ± 0.2 kcal/mol), and it also agrees with the bond energy determined previously from the CID experiment (49.5 ± 2.9 kcal/mol).

**Figure 7:**
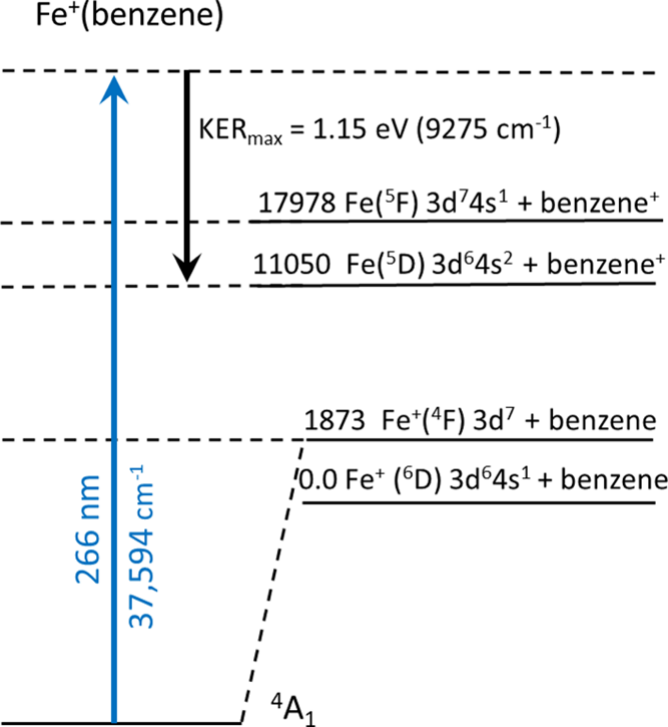
Schematic level diagram for the energy levels of Fe^+^(benzene) compared to those of the Fe^+^ atomic ion. The blue arrow shows the excitation energy used for the photofragment imaging experiment. The black downward arrow shows the maximum kinetic energy measured for the benzene^+^ photofragment.

The imaging of the benzene cation in Figure 6 has interesting implications for the dynamics of the charge-transfer process. Figure 7 shows a schematic energy level diagram including the atomic states of the iron cation and those for the neutral iron atom relevant after charge transfer. The energies of these levels are relative to that of the Fe^+^ (^6^D) + benzene asymptote. The lowest level of the respective atomic multiplets is used. The well depth of the Fe^+^(benzene) bond in this figure is placed at an energy relative to the atomic states consistent with the bond energy determined by the scanned threshold measurement [16,924 cm^–1^ or 2.10 eV below the Fe^+^ (^6^D) + benzene ground state]. As shown in the diagram, excitation from this ^4^A_1_ starting point using 266 nm carries the complex to the level indicated by the highest dotted line in the figure, and after dissociation the measured KER_max_ brings the system back down to almost exactly the level of the Fe (^5^D) + benzene^+^ ground state. This energy coincidence suggests that the KER_max_ value corresponds to the production of both the neutral iron atom and the benzene cation in their ground states. The most probable value of the KER is 0.2–0.4 eV, which is 0.9–0.7 eV (7200–5600 cm^–1^) less than the KER_max_ value. This lower KER must be balanced by internal vibrational excitation of the benzene cation or electronic excitation of the iron atom, or some of both. This energy difference coincides closely with the Fe (^5^D) – Fe (^5^F) energy difference, and so production of excited iron atoms could explain a large fraction of the reduced KER at its maximum value. Production of the 3d^7^4s^1^ (^5^F) state following charge transfer from the quartet ground state of the Fe^+^(benzene) complex makes sense electronically. The molecular complex in its quartet ground state correlates to the Fe^+^ (^4^F) 3d^7^ excited state of Fe^+^, and transfer of an electron from benzene would likely place it in the s orbital, producing the Fe (^5^F) 3d^7^4s^1^ excited state. Production of the Fe (^5^D) 3d^6^4s^2^ + benzene^+^ ground state, which apparently corresponds to the KER_max_ value, is more difficult to understand. No single-electron transition can go from the 3d^7^ configuration of Fe^+^(benzene) to the 3d^6^4s^2^ configuration Fe (^5^D) + benzene^+^, and so a more complex sequence of events (perhaps charge transfer and near-simultaneous Fe ^5^F → ^5^D fluorescence) is required to explain this. A small fraction of the benzene cation signal occurs at the KER_max_ value, and so a process like this, which might not be very efficient, could still explain that signal.

It is surprising that the upper limit on the dissociation energy of Fe^+^(benzene) determined from the imaging experiment coincides with the upper limit determined from the spectroscopic threshold and also with the dissociation energy (not an upper limit) determined by previous CID experiments. Both the scanned spectroscopic threshold and the imaging experiment give values that are upper limits, but under certain circumstances both of these values could correspond to actual dissociation energies. Because all three experiments agree with each other, it seems that this may be true. A similar situation was found recently for the Fe^+^(acetylene) complex, where CID, the scanned threshold, and photofragment imaging all produced the same dissociation energies.^68^ In the Fe^+^(acetylene) system, the dissociation energies determined (34–38 kcal/mol) were all lower than the values found here for the Fe^+^(benzene) complex. The computed values for the dissociation energies for Fe^+^(acetylene) were in poor agreement with DFT theory using B3LYP, M06-L, or MN15-L functionals. In contrast to this, the computed B3LYP value for the dissociation energy of Fe^+^(benzene) agrees reasonably well with the experiments. The values computed with other functionals, and those for the dissociation energy of Fe^+^(benzene)_2_, remain in poor agreement with the experiments. This is particularly discouraging because the problems with DFT for transition metals are well known, and the M06-L and MN15-L functionals were optimized for these systems.^95−98^

## Conclusions

Fe^+^(benzene) and Fe^+^(benzene)_2_ ions were studied with different photodissociation experiments to investigate their dissociation energies. In scanned-laser measurements, the lowest energy thresholds at which dissociation could be detected were determined. Thresholds at 532 and 591 nm for Fe^+^(benzene) and Fe^+^(benzene)_2_ led to upper limits on the adiabatic dissociation energies of 48.4 and 43.0 ± 0.2 kcal/mol, respectively. Additional experiments on Fe^+^(benzene) used photofragment imaging to measure the same dissociation energy in the charge-transfer dissociation process that produces the benzene^+^ cation. The maximum value of the KER in this process produced an upper limit on the dissociation energy of 49.3 ± 3.2 kcal/mol. The two different experimental values for the Fe^+^(benzene) complex agree with each other and with the value obtained previously by CID (49.5 ± 2.9 kcal/mol). Although the scanned threshold and imaging values for this ion are strictly-speaking upper limits on bond energies, their agreement with each other and with the previous CID results suggest that the bond energies determined may correspond to actual values. Likewise, the upper limit derived from the scanned threshold for Fe^+^(benzene)_2_ agrees with the dissociation energy determined previously for this ion with the CID of 44.7 ± 3.8 kcal/mol. This value may also correspond to an actual bond energy. The experimental values determined here for Fe^+^(benzene) all agree reasonably well with computational values determined from DFT at the B3LYP level but are in poor agreement with results obtained using other functionals. The experimental values determined for Fe^+^(benzene)_2_ are in poor agreement with all results from theory. It is reassuring that there is consensus on the dissociation energies for these transition-metal ion systems from different experiments. However, it is still discouraging that computations with DFT remain so problematic.

## Supporting Information Available

Full citation for ref (88) and the details of the DFT computations done, including the structures and energetics for each of the cluster ions considered (PDF)
