# Correction to “Photodissociation Spectroscopy and Photofragment Imaging to Probe Fe^+^(Benzene)_1,2_ Dissociation Energies”

**DOI:** 10.1021/acs.jpca.4c03633

**Published:** 2024-06-20

**Authors:** Jason E. Colley, Nathan J. Dynak, John R. C. Blais, Michael A. Duncan

In this paper we reported bond dissociation energies determined from the observed thresholds for photodissociation. Although strictly speaking the photodissociation thresholds represent upper limits on the bond energies, we argued that these complexes have near-continuous densities of states and that the thresholds should be interpreted as the actual bond energies.

In the analysis of the threshold for Fe^+^(benzene) we noted that the ground state had a quartet spin and that the excited state accessed optically should be one with this same quartet spin. However, the ground state dissociation products should be Fe^+^ (^6^D) 3d^6^4s^1^ + benzene. We therefore applied a correction corresponding to the energy of the lowest energy quartet state for Fe^+^ (^4^F) 3d^7^ to obtain the adiabatic dissociation energy. The correction subtracted the energy of the ^4^F state (1873 cm^–1^) from the observed photodissociation threshold energy (532 ± 2 nm; 18,797 cm^–1^) to obtain the adiabatic dissociation energy (16,924 cm^–1^; 48.4 ± 0.2 kcal/mol). This original procedure is correct for the Fe^+^(benzene) complex.

Our mistake was to apply this same procedure to the Fe^+^(benzene)_2_ complex. We have now recognized that no correction for asymptotic spin states is required for this system. The ground state of Fe^+^(benzene)_2_ is a quartet, and optical excitation should also access quartet excited states. The dissociation threshold produces the Fe^+^(benzene) + benzene with the Fe^+^(benzene) in its ground quartet state. The dissociation threshold reported at 591 ± 2 nm therefore provides the bond energy directly (16,921 cm^–1^; 48.4 ± 0.2 kcal/mol), with no spin-state correction required. The originally reported dissociation energy for Fe^+^(benzene)_2_ of 43.0 ± 0.2 kcal/mol, which included a spin-state correction, is therefore incorrect and the actual value is 48.4 ± 0.2 kcal/mol.

