# Supplementary material for: Photodissociation Spectroscopy and Photofragment Imaging to Probe Fe+(Benzene)1,2 Dissociation Energies
Source: J Phys Chem A. 2023 Mar 15;127(12):2795–804. doi: 10.1021/acs.jpca.3c00735 (PMC10068738; doi:10.1021/acs.jpca.3c00735)
Supplement: Supplementary file 1 — jp3c00735_si_001.pdf [file jp3c00735_si_001.pdf]

**Supporting Information:**

# *Photodissociation Spectroscopy and Photofragment Imaging to Probe $Fe^+(Benzene)_{1,2}$ Dissociation Energies*

Jason E. Colley, Nathan J. Dynak, John R. C. Blais, Michael A. Duncan*

Department of Chemistry, University of Georgia, Athens, Georgia 30602, United States

*Email: maduncan@uga.edu

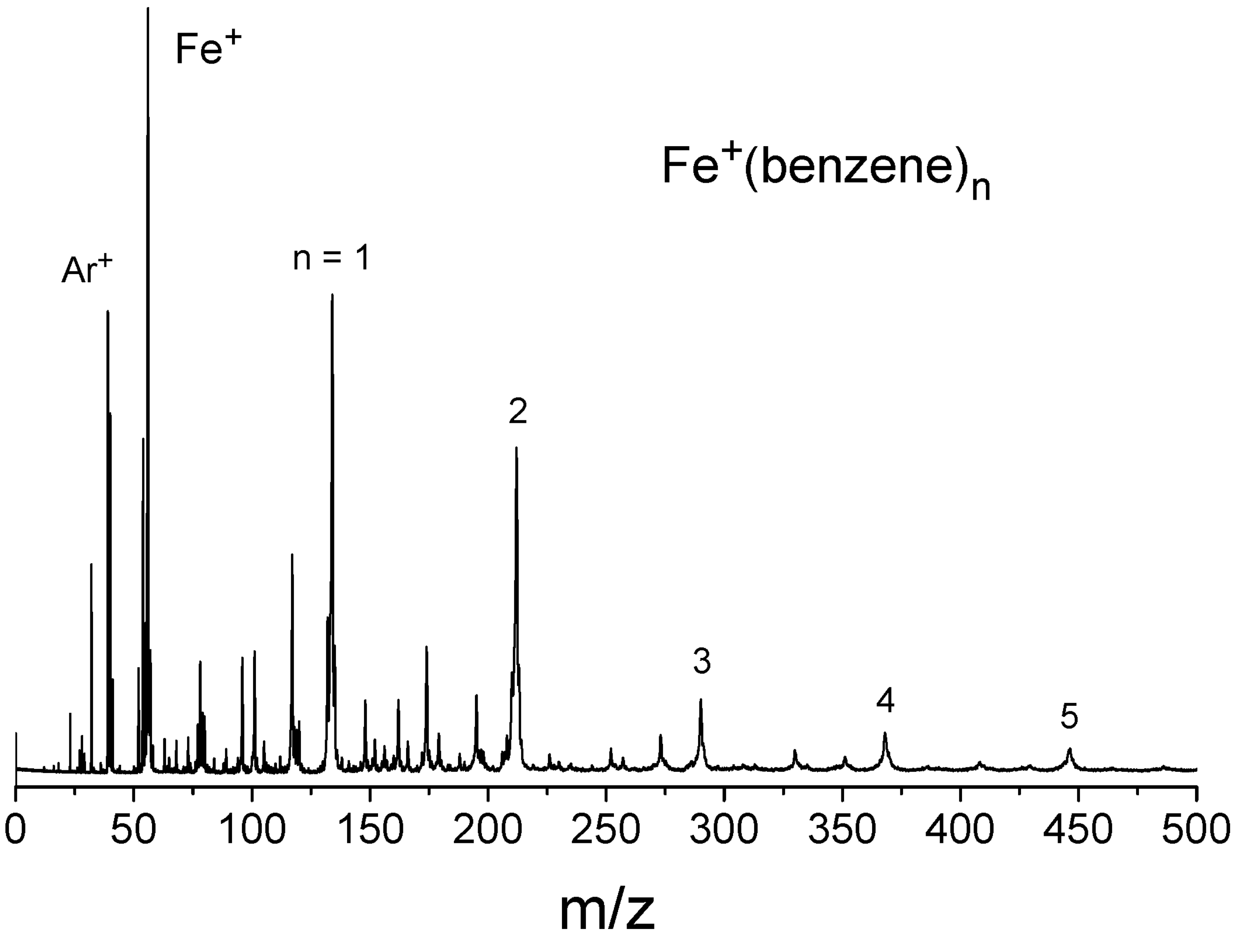


Figure S1. Mass spectrum of iron cation-benzene complexes produced by laser vaporization. A laser pulse energy of 8 mJ/pulse at 355 nm was used for the laser vaporization.

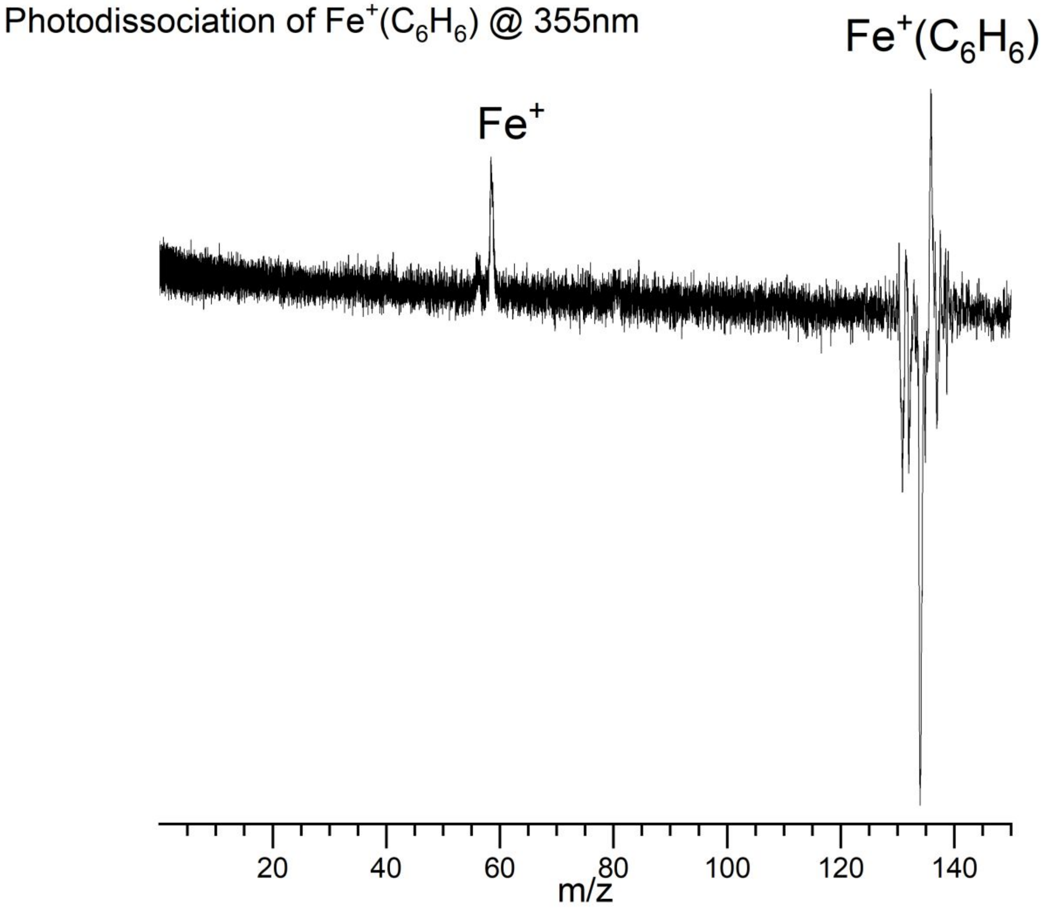


Figure S2. Photodissociation mass spectrum of $Fe^{+}$(benzene) at 355 nm which produces only the $Fe^{+}$ photofragment.

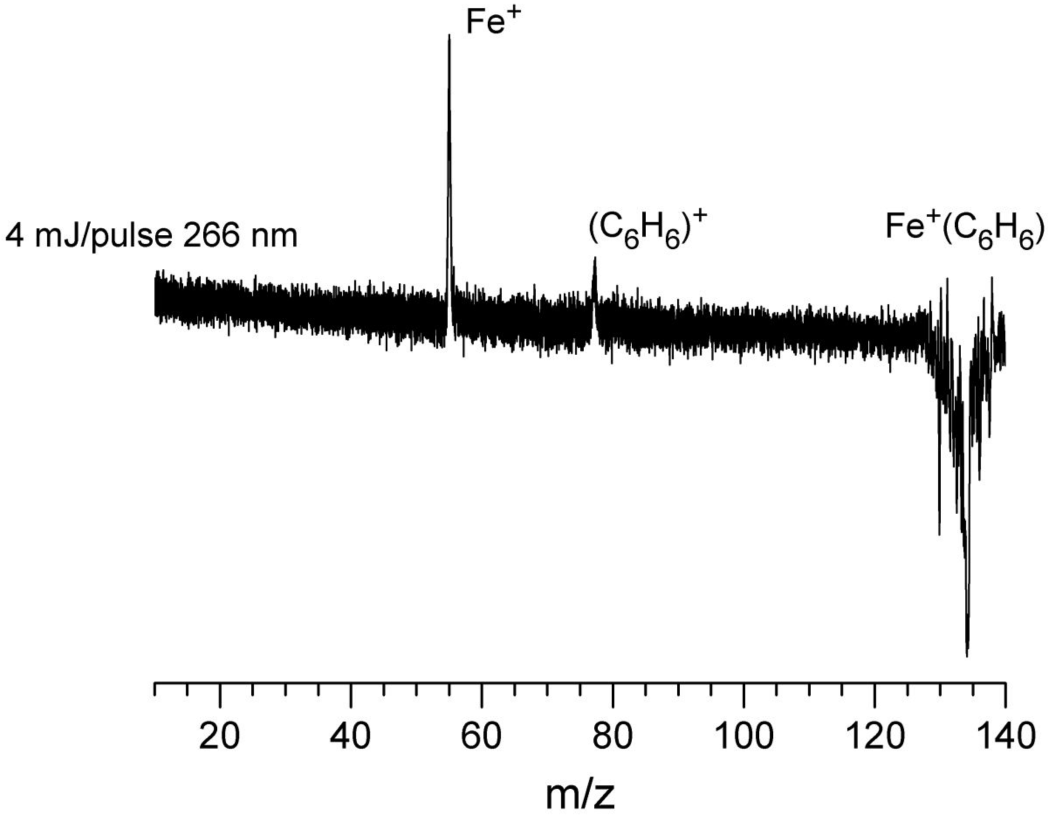


Figure S3. Photodissociation mass spectrum of $Fe^+$(benzene) at 266 nm, which produces both the $Fe^+$ and benzene$^+$ photofragments.

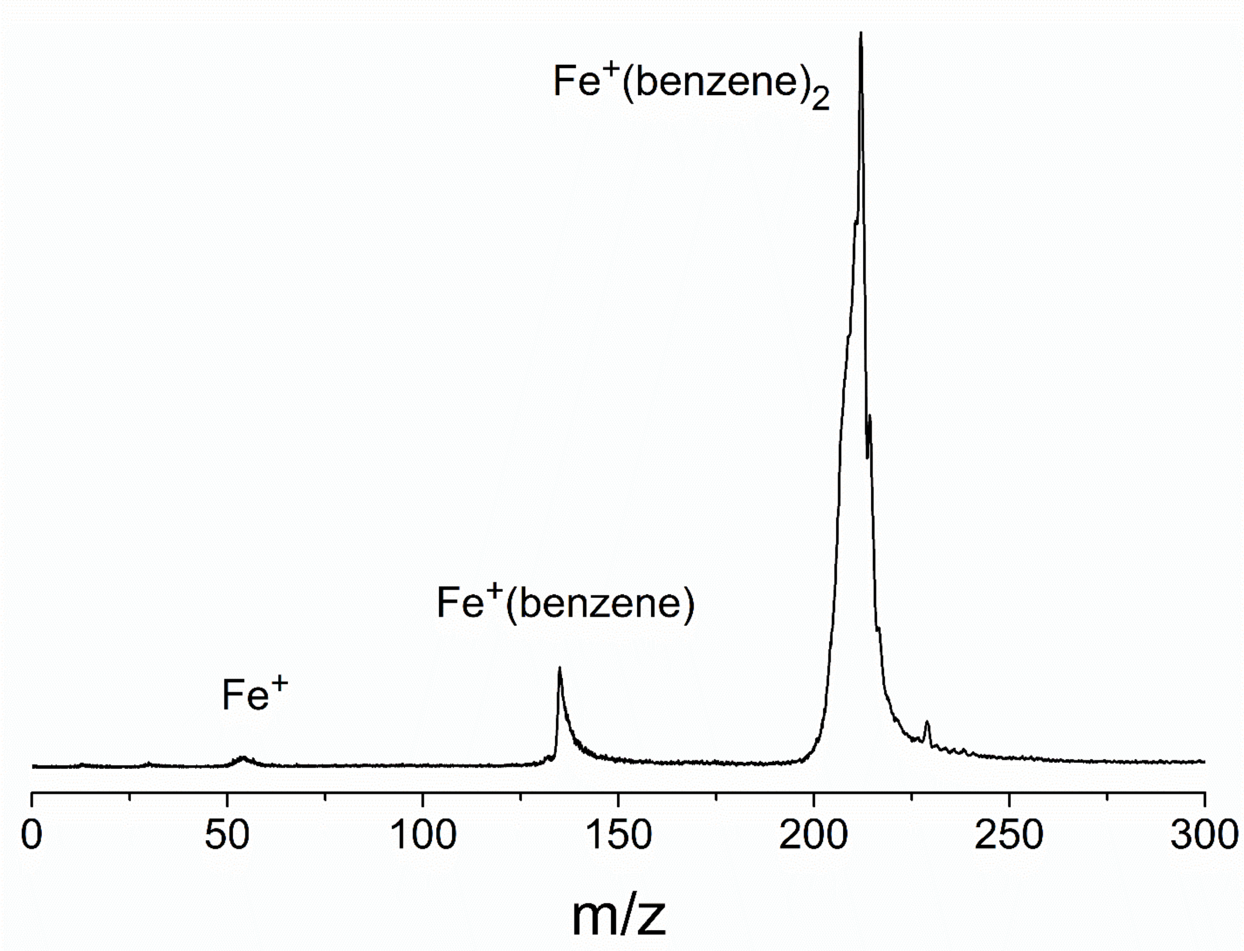


Figure S4. Photodissociation mass spectrum of $Fe^{+}$(benzene)$_2$ which produces primarily the $Fe^{+}$(benzene) photofragment. 2 mJ/pulse at 260 nm was used for the photodissociation laser.

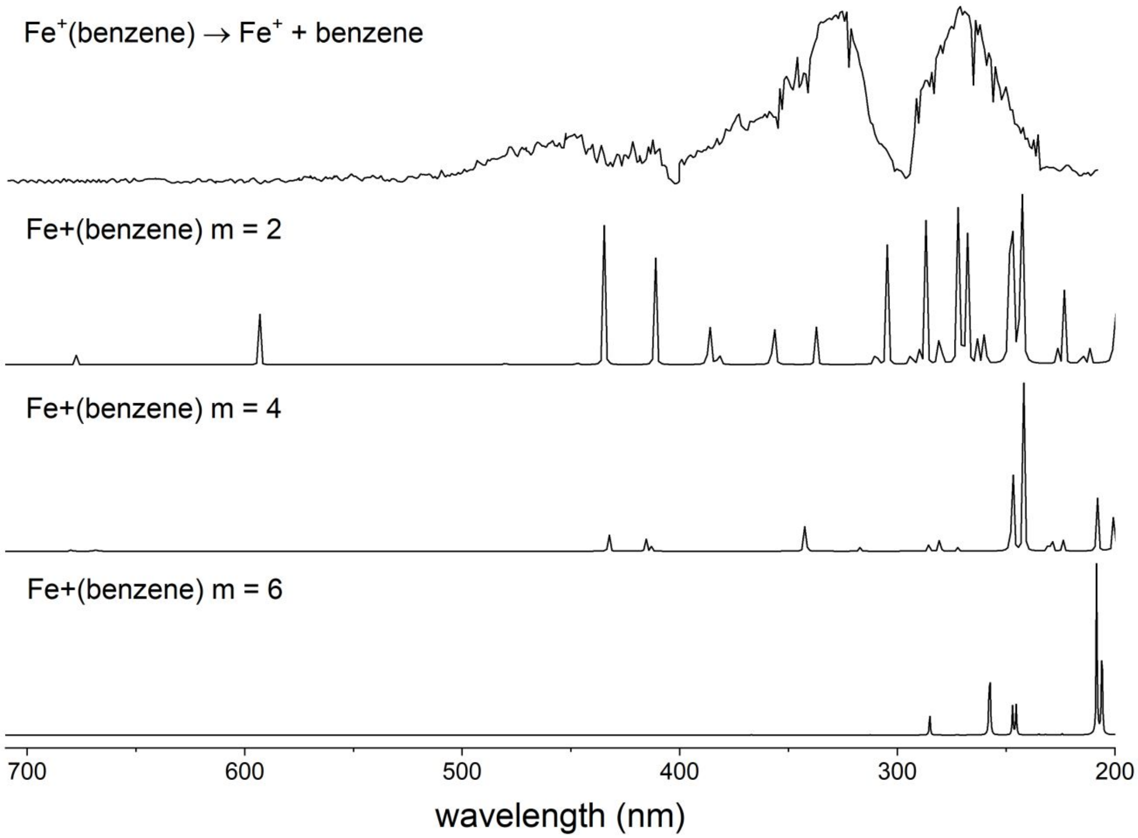


Figure S5. The photodissociation spectrum of $Fe^+$(benzene) → $Fe^+$ + benzene in the 700–200 nm region compared to the predictions of theory for ions in different spin states.

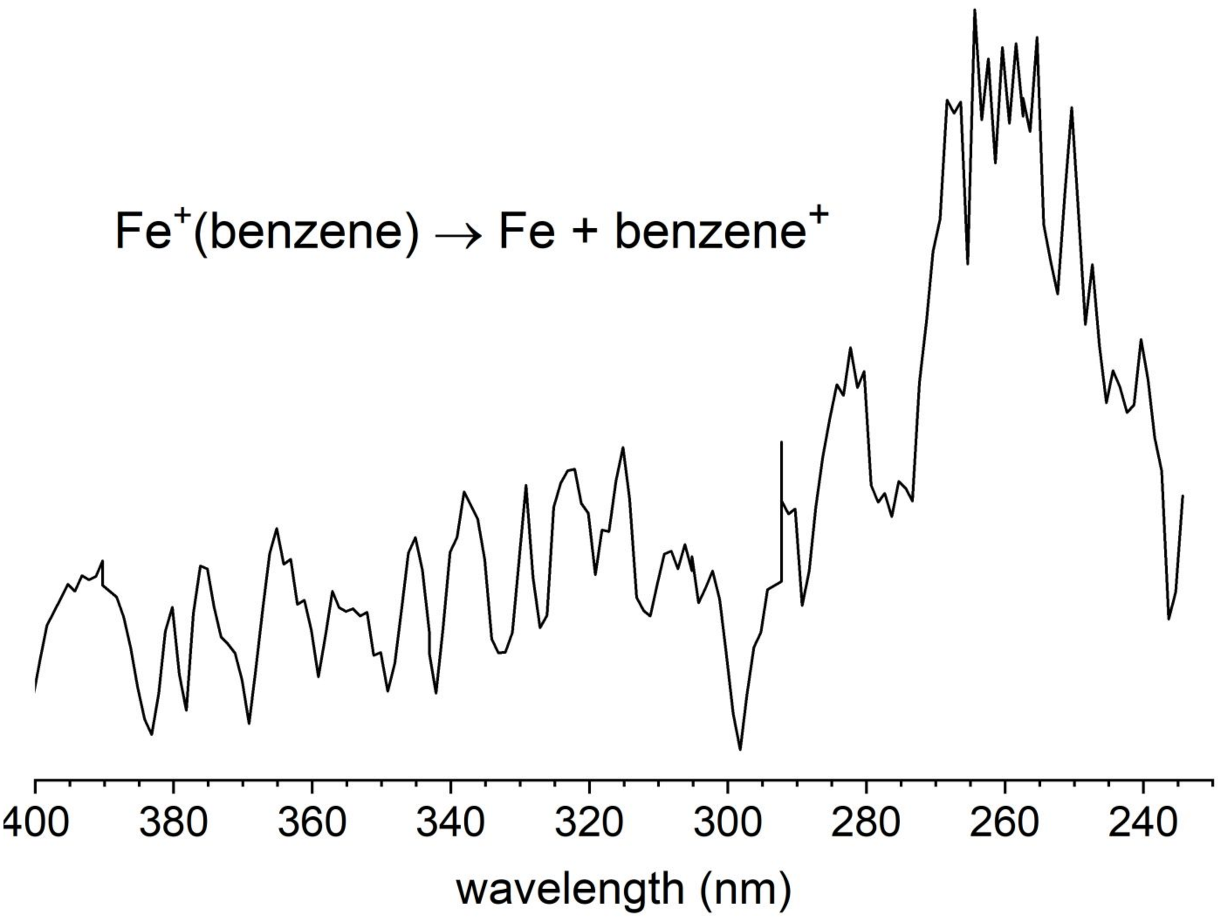


Figure S6. The photodissociation spectrum of $Fe^{+}$(benzene) → Fe + $benzene^{+}$ in the 400–230 nm region.

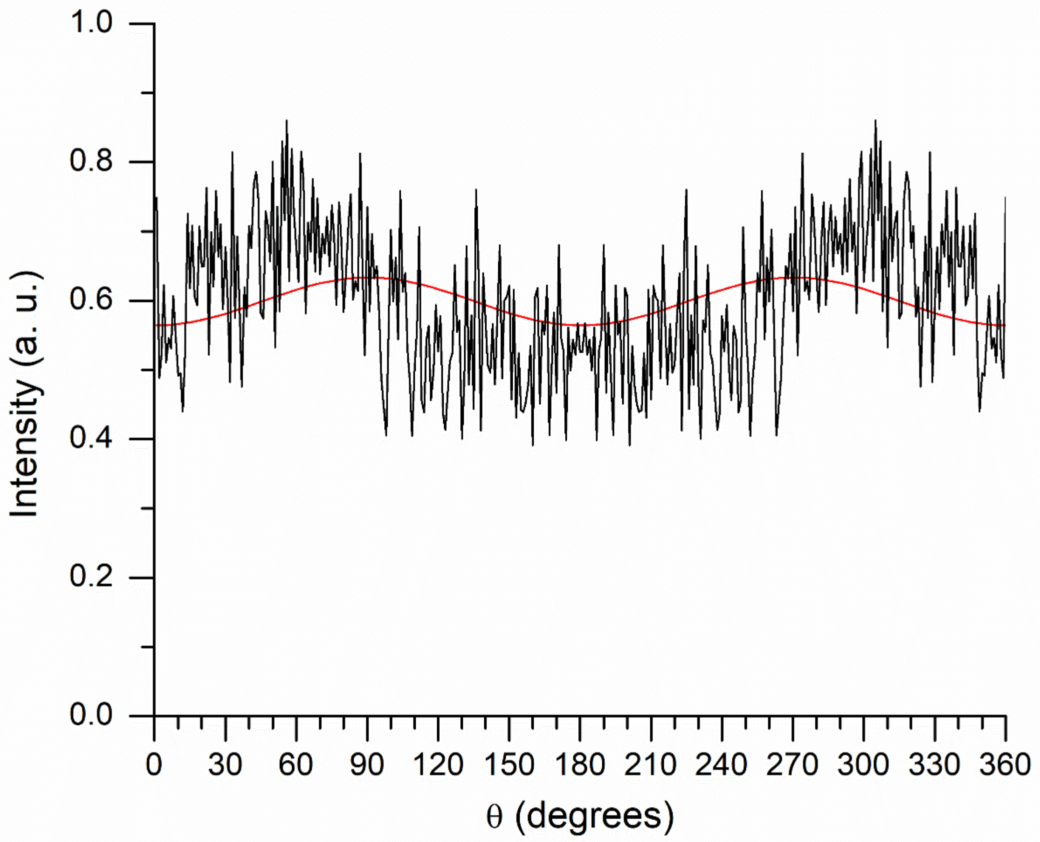


Figure S7. The angular distribution and fit of the $Fe^+$ photofragment image from $Fe^+$(benzene) photodissociation at 266 nm. The red line is a fit with $\beta = -0.08$.

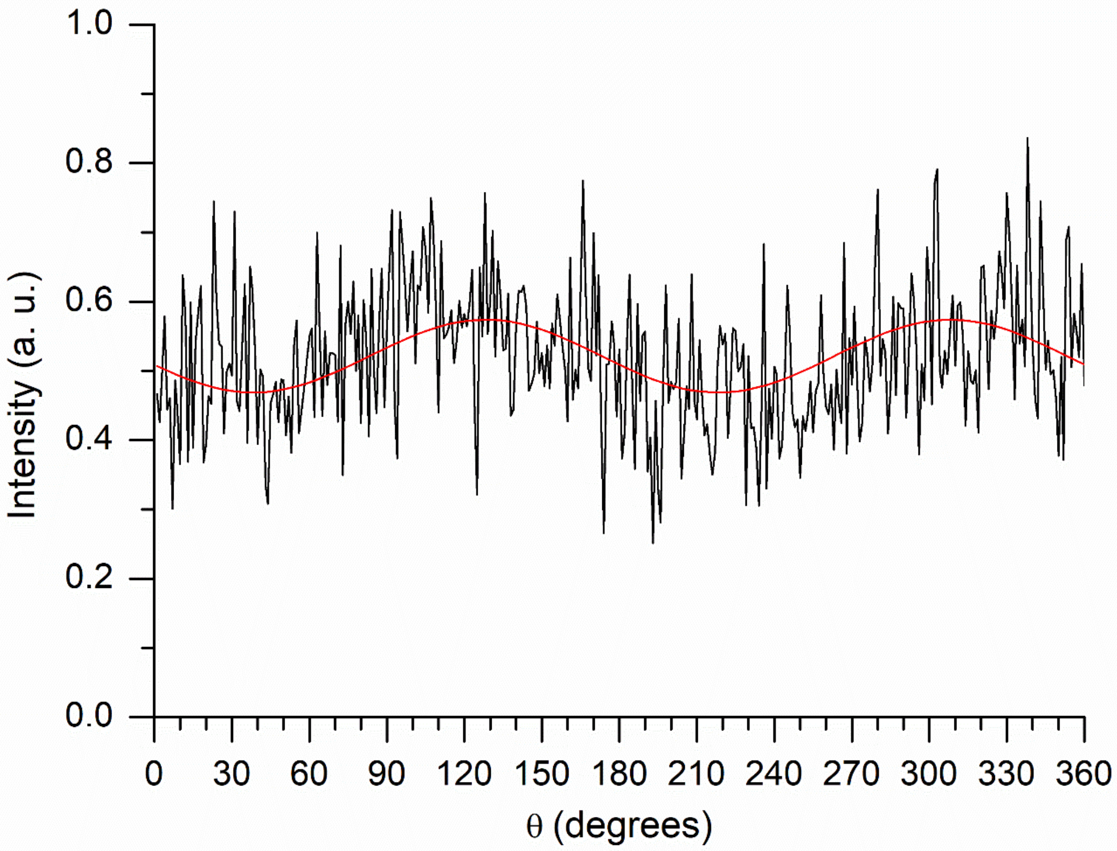


Figure S8. The angular distribution of the benzene cation photofragment image from the dissociation of $Fe^{+}$(benzene) at 266 nm. The red line is a fit with $\beta = -0.13$.

Full citation for reference 88:

Frisch, M. J.; Trucks, G. W.; Schlegel, H. B.; Scuseria, G. E.; Robb, M. A.; Cheeseman, J. R.; Scalmani, G.; Barone, V.; Peterson, G. A.; Nakatsuji, H.; Li, X.; Caricato, M.; Marenich, A. V.; Bloino, J.; Janesko, B. G.; Gomperts, R.; Mennucci, B.; Hratchian, H. P.; Ortiz, J. V.; Izmaylov, A. F.; Sonnenberg, J. L.; Williams-Young, D.; Ding, F.; Lipparini, F.; Egidi, F.; Goings, J.; Peng, B.; Petrone, A.; Henderson, T.; Ranasinghe, D.; Zakrzewski, V. G.; Gao, J.; Rega, N.; Zheng, G.; Liang, W.; Hada, M.; Ehara, M.; Toyota, K.; Fukuda, R.; Hasegawa, J.; Ishida, M.; Nakajima, T.; Honda, Y.; Kitao, O.; Nakai, H.; Vreven, T.; Throssell, K.; Montgomery, Jr, J. A.; Peralta, E.; Ogliaro, F.; Bearpark, M. J.; Heyd, J. J.; Brothers, E. N.; Kudin, K. N.; Staroverov, V. N.; Keith, T. A.; Kobayashi, R.; Normand, J.; Raghavachari, K.; Rendell, A. P.; Burant, J. C.; Iyengar, S. S.; Tomasi, J.; Cossi, M.; Millam, J. M.; Klene, M.; Adamo, C.; Cammi, R.; Ochterski, J. W.; Martin, R. L.; Morokuma, K.; Farkas, O.; Foresman, J. B.; Fox, D. J. Gaussian 16 (Revision C.01) Gaussian, Inc., Wallingford CT, 2019.

---

All calculations were carried out using an “ultrafine” integration grid, and the optimization threshold for energy and structure optimizations were set to “tight.” The “stable=opt” keyword was used on all structures to check for electronic wavefunction stability. All electronic energies are corrected for zero-point vibrational energy. The first 100 electronic transitions were calculated using TD-DFT.

$Fe^{+}$
B3LYP
m = 2
Electronic Transitions

| Wavelength, nm | Oscillator strength |
|---|---|
| 7044.33 | 0.0000 |
| 5814.08 | 0.0000 |
| 5788.78 | 0.0000 |
| 3193.23 | 0.0000 |
| 3188.79 | 0.0000 |
| 1628.88 | 0.0000 |
| 1627.47 | 0.0000 |
| 1558.19 | 0.0000 |
| 1557.82 | 0.0000 |
| 955.64 | 0.0000 |
| 932.10 | 0.0000 |
| 931.96 | 0.0000 |
| 645.36 | 0.0000 |
| 571.16 | 0.0000 |
| 571.07 | 0.0000 |
| 508.88 | 0.0000 |
| 508.86 | 0.0000 |
| 215.84 | 0.0025 |
| 215.80 | 0.0025 |
| 211.41 | 0.0013 |
| 204.63 | 0.0001 |
| 204.59 | 0.0002 |
| 203.65 | 0.0000 |
| 198.24 | 0.0000 |
| 195.69 | 0.0107 |
| 192.19 | 0.0015 |
| 192.17 | 0.0015 |
| 188.14 | 0.0378 |
| 188.13 | 0.0378 |
| 182.20 | 0.0151 |
| 174.53 | 0.0000 |
| 174.52 | 0.0000 |
| 169.30 | 0.0000 |
| 167.67 | 0.0070 |
| 167.67 | 0.0071 |
| 164.08 | 0.1300 |
| 163.60 | 0.1070 |
| 163.58 | 0.1068 |
| 124.98 | 0.0000 |
| 124.97 | 0.0000 |
| 120.36 | 0.0000 |

| | |
|---|---|
| 120.36 | 0.0000 |
| 113.80 | 0.0000 |
| 113.50 | 0.0000 |
| 112.88 | 0.0000 |
| 112.87 | 0.0000 |
| 112.25 | 0.0000 |
| 112.25 | 0.0000 |
| 111.10 | 0.0000 |
| 110.00 | 0.0000 |
| 110.00 | 0.0000 |
| 108.57 | 0.0000 |
| 108.32 | 0.0000 |
| 108.31 | 0.0000 |
| 108.06 | 0.0000 |
| 107.86 | 0.0000 |
| 107.85 | 0.0000 |
| 107.72 | 0.0000 |
| 107.48 | 0.0000 |
| 107.45 | 0.0000 |
| 107.31 | 0.0000 |
| 106.79 | 0.0000 |
| 105.07 | 0.0000 |
| 104.46 | 0.0000 |
| 104.45 | 0.0000 |
| 103.06 | 0.0000 |
| 103.06 | 0.0000 |
| 102.92 | 0.0000 |

$Fe^+$
B3LYP
m = 4
Electronic Transitions

| Wavelength, nm | Oscillator strength |
|---|---|
| 6986.2 | 0.0000 |
| 6918.55 | 0.0000 |
| 6824.61 | 0.0000 |
| 3206.65 | 0.0000 |
| 3176.99 | 0.0000 |
| 1050.45 | 0.0000 |
| 1050.00 | 0.0000 |
| 1049.73 | 0.0000 |
| 805.02 | 0.0000 |
| 804.98 | 0.0000 |
| 511.15 | 0.0000 |
| 511.14 | 0.0000 |
| 511.14 | 0.0000 |
| 226.64 | 0.0149 |
| 226.54 | 0.0149 |
| 226.51 | 0.0149 |
| 220.91 | 0.0000 |
| 220.87 | 0.0000 |
| 220.78 | 0.0000 |
| 184.99 | 0.0000 |
| 184.98 | 0.0000 |
| 184.98 | 0.0000 |
| 183.84 | 0.0282 |
| 183.84 | 0.0282 |
| 183.83 | 0.0282 |
| 166.80 | 0.0000 |
| 163.86 | 0.0000 |
| 163.86 | 0.0000 |
| 163.86 | 0.0000 |
| 160.18 | 0.0000 |
| 160.17 | 0.0000 |
| 157.71 | 0.1129 |
| 157.71 | 0.1129 |
| 157.71 | 0.1129 |
| 132.70 | 0.0000 |
| 132.67 | 0.0000 |
| 120.16 | 0.0000 |
| 120.15 | 0.0000 |
| 118.12 | 0.0000 |
| 118.06 | 0.0000 |
| 115.73 | 0.0000 |

| | |
|---|---|
| 115.73 | 0.0000 |
| 114.45 | 0.0000 |
| 114.42 | 0.0000 |
| 114.38 | 0.0000 |
| 113.30 | 0.0000 |
| 113.25 | 0.0000 |
| 113.23 | 0.0000 |
| 106.69 | 0.0000 |
| 106.69 | 0.0000 |
| 106.69 | 0.0000 |
| 103.86 | 0.0000 |
| 103.86 | 0.0000 |
| 103.86 | 0.0000 |
| 103.78 | 0.0000 |
| 103.78 | 0.0000 |
| 103.04 | 0.0000 |
| 103.04 | 0.0000 |
| 103.04 | 0.0000 |
| 102.79 | 0.0000 |
| 101.91 | 0.0000 |

$Fe^{+}$
B3LYP
m = 6
Electronic Transitions

| Wavelength, nm | Oscillator strength |
|---|---|
| 18169.58 | 0.0000 |
| 8534.06 | 0.0000 |
| 8409.96 | 0.0000 |
| 5517.95 | 0.0000 |
| 395.63 | 0.0000 |
| 218.55 | 0.2977 |
| 208.93 | 0.2678 |
| 208.92 | 0.2680 |
| 147.79 | 0.0000 |
| 147.31 | 0.0357 |
| 147.12 | 0.0353 |
| 134.24 | 0.0000 |
| 128.33 | 0.0371 |
| 128.32 | 0.0372 |
| 123.97 | 0.0000 |
| 121.29 | 0.0000 |
| 116.56 | 0.0276 |
| 116.55 | 0.0276 |
| 115.42 | 0.0000 |
| 115.02 | 0.0000 |
| 115.02 | 0.0000 |
| 113.79 | 0.0000 |
| 113.07 | 0.0000 |
| 112.48 | 0.0692 |
| 112.48 | 0.0692 |
| 111.71 | 0.0029 |
| 109.94 | 0.0000 |
| 109.91 | 0.0000 |
| 108.62 | 0.0000 |
| 106.8 | 0.1338 |
| 106.68 | 0.0364 |
| 106.68 | 0.0364 |

$Fe^+$

M06-L

m = 2

Electronic Transitions

| Wavelength, nm | Oscillator strength |
|---|---|
| 3662.44 | 0.0000 |
| 3610.80 | 0.0000 |
| 3341.25 | 0.0000 |
| 2395.26 | 0.0000 |
| 2362.23 | 0.0000 |
| 1716.21 | 0.0000 |
| 1571.17 | 0.0000 |
| 1118.12 | 0.0000 |
| 879.54 | 0.0000 |
| 833.60 | 0.0000 |
| 785.05 | 0.0000 |
| 772.27 | 0.0000 |
| 741.62 | 0.0000 |
| 629.66 | 0.0000 |
| 497.97 | 0.0000 |
| 425.68 | 0.0000 |
| 394.55 | 0.0000 |
| 238.66 | 0.0018 |
| 219.42 | 0.0040 |
| 218.37 | 0.0447 |
| 216.67 | 0.0124 |
| 216.45 | 0.0001 |
| 209.49 | 0.0003 |
| 200.42 | 0.0238 |
| 197.74 | 0.0105 |
| 195.47 | 0.0422 |
| 192.76 | 0.0002 |
| 186.25 | 0.0000 |
| 183.77 | 0.0109 |
| 182.88 | 0.0387 |
| 180.31 | 0.0000 |
| 173.45 | 0.0211 |
| 171.01 | 0.1163 |
| 167.72 | 0.1007 |
| 164.19 | 0.0000 |
| 162.27 | 0.0599 |
| 154.46 | 0.0006 |
| 153.71 | 0.0014 |
| 140.07 | 0.0000 |
| 139.46 | 0.0000 |
| 134.25 | 0.0000 |

| | |
|---|---|
| 131.01 | 0.0000 |
| 123.43 | 0.0000 |
| 123.38 | 0.0000 |
| 119.05 | 0.0000 |
| 118.85 | 0.0000 |
| 116.76 | 0.0000 |
| 114.65 | 0.0000 |
| 114.36 | 0.0000 |
| 114.10 | 0.0000 |
| 113.37 | 0.0000 |
| 112.71 | 0.0000 |
| 112.35 | 0.0000 |
| 112.03 | 0.0000 |
| 111.91 | 0.0000 |
| 111.91 | 0.0000 |
| 110.86 | 0.0000 |
| 110.49 | 0.0000 |
| 110.39 | 0.0000 |
| 109.84 | 0.0000 |
| 109.43 | 0.0000 |
| 108.63 | 0.0000 |
| 108.00 | 0.0000 |
| 107.38 | 0.0000 |
| 106.40 | 0.0000 |
| 105.41 | 0.0000 |
| 103.86 | 0.0000 |
| 103.41 | 0.0000 |
| 103.36 | 0.0000 |
| 102.91 | 0.0000 |
| 102.56 | 0.0000 |
| 102.55 | 0.0000 |
| 102.39 | 0.0000 |
| 101.15 | 0.0000 |
| 101.01 | 0.0000 |
| 100.53 | 0.0000 |

$Fe^{+}$
M06-L
m = 4
Electronic Transitions could not be calculated

$Fe^{+}$
M06-L
m = 6
Electronic Transitions

| Wavelength, nm | Oscillator strength |
|---|---|
| 4266.08 | 0.0000 |
| 4175.56 | 0.0000 |
| 2842.31 | 0.0000 |
| 2096.23 | 0.0000 |
| 388.36 | 0.0000 |
| 231.66 | 0.3204 |
| 218.22 | 0.2799 |
| 217.83 | 0.2853 |
| 151.99 | 0.0622 |
| 147.85 | 0.0543 |
| 132.55 | 0.0000 |
| 127.30 | 0.0265 |
| 127.26 | 0.0275 |
| 123.35 | 0.0000 |
| 121.33 | 0.0000 |
| 119.30 | 0.0000 |
| 118.71 | 0.0000 |
| 118.70 | 0.0000 |
| 116.53 | 0.0000 |
| 116.34 | 0.0250 |
| 116.33 | 0.0251 |
| 115.38 | 0.0000 |
| 112.97 | 0.0669 |
| 112.95 | 0.0678 |
| 112.10 | 0.0045 |
| 109.64 | 0.0000 |
| 109.50 | 0.0000 |
| 107.74 | 0.0000 |
| 107.37 | 0.0000 |
| 106.97 | 0.1180 |
| 106.17 | 0.0280 |
| 106.17 | 0.0283 |

$Fe^{+}$
MN15-L
m = 2
Electronic Transitions

| Wavelength, nm | Oscillator strength |
|---|---|
| 5208.49 | 0.0000 |
| 4533.25 | 0.0000 |
| 3950.33 | 0.0000 |
| 2261.58 | 0.0000 |
| 2171.70 | 0.0000 |
| 1089.65 | 0.0000 |
| 1016.04 | 0.0000 |
| 912.72 | 0.0000 |
| 888.90 | 0.0000 |
| 766.58 | 0.0000 |
| 637.17 | 0.0000 |
| 622.97 | 0.0000 |
| 561.77 | 0.0000 |
| 548.68 | 0.0000 |
| 529.79 | 0.0000 |
| 404.32 | 0.0000 |
| 401.69 | 0.0000 |
| 227.86 | 0.0473 |
| 224.83 | 0.0242 |
| 215.72 | 0.0092 |
| 209.50 | 0.0465 |
| 197.88 | 0.0367 |
| 196.82 | 0.0105 |
| 188.01 | 0.0000 |
| 182.48 | 0.0044 |
| 182.43 | 0.0202 |
| 180.87 | 0.0010 |
| 180.44 | 0.0069 |
| 176.74 | 0.0006 |
| 169.19 | 0.0382 |
| 169.12 | 0.0407 |
| 165.81 | 0.0346 |
| 163.30 | 0.0759 |
| 158.07 | 0.1326 |
| 157.45 | 0.0737 |
| 156.91 | 0.0064 |
| 153.69 | 0.0013 |
| 151.15 | 0.0021 |
| 136.62 | 0.0000 |
| 135.06 | 0.0000 |
| 126.91 | 0.0000 |

| | |
|---|---|
| 122.36 | 0.0000 |
| 121.08 | 0.0000 |
| 121.07 | 0.0000 |
| 120.24 | 0.0000 |
| 118.70 | 0.0000 |
| 118.66 | 0.0000 |
| 118.56 | 0.0000 |
| 118.42 | 0.0000 |
| 118.12 | 0.0000 |
| 116.53 | 0.0000 |
| 116.44 | 0.0000 |
| 114.57 | 0.0000 |
| 114.55 | 0.0000 |
| 114.21 | 0.0000 |
| 111.46 | 0.0000 |
| 109.72 | 0.0000 |
| 109.59 | 0.0000 |
| 108.50 | 0.0000 |
| 107.93 | 0.0000 |
| 106.91 | 0.0000 |
| 106.53 | 0.0000 |
| 106.21 | 0.0000 |
| 105.91 | 0.0000 |
| 105.44 | 0.0000 |
| 105.27 | 0.0000 |
| 105.10 | 0.0000 |
| 104.49 | 0.0000 |
| 104.09 | 0.0000 |
| 103.15 | 0.0000 |
| 102.27 | 0.0000 |
| 102.22 | 0.0000 |
| 101.88 | 0.0000 |
| 101.30 | 0.0000 |
| 101.27 | 0.0000 |
| 101.02 | 0.0000 |
| 101.01 | 0.0000 |
| 100.68 | 0.0000 |
| 100.23 | 0.0000 |

$Fe^{+}$
MN15-L
m = 4
Electronic Transitions

| Wavelength, nm | Oscillator strength |
|---|---|
| 7291.92 | 0.0000 |
| 5924.31 | 0.0000 |
| 5916.87 | 0.0000 |
| 1880.17 | 0.0000 |
| 1190.72 | 0.0000 |
| 1184.19 | 0.0000 |
| 1183.96 | 0.0000 |
| 817.93 | 0.0000 |
| 349.18 | 0.0000 |
| 322.78 | 0.0000 |
| 285.85 | 0.0000 |
| 285.84 | 0.0000 |
| 282.86 | 0.0000 |
| 246.49 | 0.0419 |
| 246.48 | 0.0417 |
| 233.48 | 0.1731 |
| 233.48 | 0.1729 |
| 197.93 | 0.0499 |
| 189.36 | 0.0000 |
| 144.54 | 0.0000 |
| 142.25 | 0.0028 |
| 142.25 | 0.0028 |
| 140.80 | 0.0000 |
| 140.60 | 0.0000 |
| 139.29 | 0.0000 |
| 137.05 | 0.0027 |
| 137.05 | 0.0027 |
| 136.87 | 0.0138 |
| 129.95 | 0.1333 |
| 129.95 | 0.1333 |
| 127.49 | 0.0000 |
| 126.95 | 0.0000 |
| 126.93 | 0.0000 |
| 126.40 | 0.0000 |
| 126.22 | 0.0000 |
| 125.55 | 0.0000 |
| 125.08 | 0.1242 |
| 122.35 | 0.0146 |
| 122.35 | 0.0146 |
| 122.07 | 0.0000 |
| 120.83 | 0.0000 |

| | |
|---|---|
| 119.71 | 0.0000 |
| 119.71 | 0.0000 |
| 119.06 | 0.0000 |
| 118.53 | 0.0000 |
| 117.35 | 0.0000 |
| 104.27 | 0.0000 |
| 102.11 | 0.0000 |

$Fe^{+}$
MN15-L
m = 6
Electronic Transitions

| Wavelength, nm | Oscillator strength |
|---|---|
| 5029.61 | 0.0000 |
| 4327.66 | 0.0000 |
| 4308.69 | 0.0000 |
| 1406.32 | 0.0000 |
| 539.94 | 0.0000 |
| 246.12 | 0.3554 |
| 231.18 | 0.2760 |
| 231.17 | 0.2761 |
| 177.34 | 0.1183 |
| 177.31 | 0.1181 |
| 151.01 | 0.0000 |
| 128.13 | 0.0000 |
| 122.20 | 0.0000 |
| 119.70 | 0.0063 |
| 119.70 | 0.0063 |
| 118.84 | 0.0000 |
| 117.74 | 0.0000 |
| 117.70 | 0.0000 |
| 117.70 | 0.0000 |
| 116.75 | 0.0000 |
| 116.47 | 0.0000 |
| 114.75 | 0.0000 |
| 114.24 | 0.0533 |
| 114.24 | 0.0533 |
| 109.73 | 0.0427 |
| 109.73 | 0.0427 |
| 107.98 | 0.0012 |
| 105.95 | 0.0000 |
| 105.71 | 0.0000 |
| 105.67 | 0.0000 |
| 104.09 | 0.1096 |
| 103.19 | 0.0000 |
| 101.54 | 0.0182 |
| 101.54 | 0.0182 |

$Fe^+$(benzene)
B3LYP
m = 2

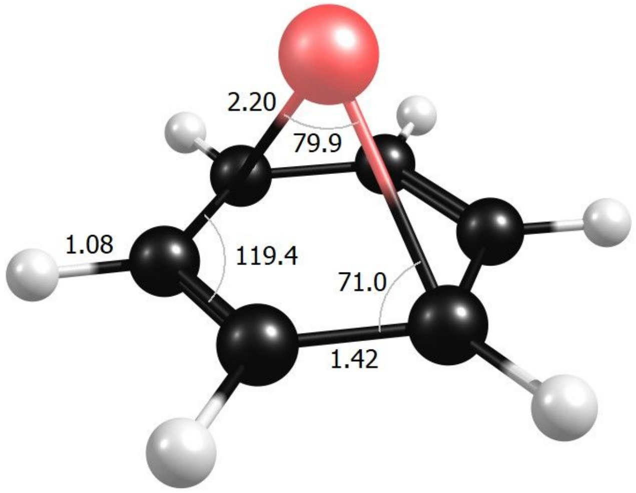


Coordinates:

| | | | |
|---|---|---|---|
| Fe | -0.000103000 | -0.064612000 | 1.047011000 |
| C | 1.417137000 | 0.042541000 | -0.688690000 |
| C | 0.709053000 | -1.174874000 | -0.693974000 |
| C | 0.711454000 | 1.250479000 | -0.566513000 |
| H | 1.243985000 | -2.114535000 | -0.720001000 |
| H | 1.247191000 | 2.186815000 | -0.489103000 |
| C | -0.710660000 | -1.173873000 | -0.694203000 |
| C | -0.709603000 | 1.251470000 | -0.566418000 |
| H | -1.246914000 | -2.112777000 | -0.720364000 |
| H | -1.244038000 | 2.188549000 | -0.489028000 |
| C | -1.416998000 | 0.044524000 | -0.688820000 |
| H | -2.498587000 | 0.046899000 | -0.706131000 |
| H | 2.498729000 | 0.043365000 | -0.705945000 |

Electronic Transitions

| Wavelength, nm | Oscillator strength |
|---|---|
| 3600.06 | 0.0000 |
| 2743.82 | 0.0000 |
| 1691.42 | 0.0001 |
| 1444.83 | 0.0001 |
| 1271.76 | 0.0005 |
| 1193.80 | 0.0008 |
| 894.81 | 0.0000 |
| 741.48 | 0.0010 |
| 677.63 | 0.0003 |
| 593.14 | 0.0010 |
| 480.06 | 0.0002 |

| | |
|---|---|
| 447.31 | 0.0002 |
| 434.72 | 0.0034 |
| 411.34 | 0.0030 |
| 386.50 | 0.0033 |
| 382.15 | 0.0010 |
| 356.95 | 0.0035 |
| 337.39 | 0.0011 |
| 309.91 | 0.0013 |
| 304.59 | 0.0026 |
| 293.69 | 0.0011 |
| 289.97 | 0.0003 |
| 287.00 | 0.0031 |
| 280.41 | 0.0030 |
| 271.83 | 0.0074 |
| 268.22 | 0.0019 |
| 267.93 | 0.0042 |
| 263.18 | 0.0005 |
| 259.84 | 0.0024 |
| 252.08 | 0.0000 |
| 247.88 | 0.0116 |
| 247.40 | 0.0065 |
| 244.72 | 0.0009 |
| 242.97 | 0.0128 |
| 226.53 | 0.0006 |
| 225.43 | 0.0000 |
| 223.23 | 0.0016 |
| 217.19 | 0.0000 |
| 216.39 | 0.0002 |
| 214.86 | 0.0005 |
| 211.53 | 0.0003 |
| 200.11 | 0.0042 |
| 196.84 | 0.0003 |
| 196.58 | 0.0001 |
| 191.10 | 0.0019 |
| 186.92 | 0.0069 |
| 186.68 | 0.0000 |
| 186.64 | 0.0130 |
| 184.71 | 0.0262 |
| 180.14 | 0.0047 |
| 176.80 | 0.0001 |
| 176.67 | 0.0000 |
| 175.59 | 0.0000 |
| 174.89 | 0.0043 |
| 173.37 | 0.0007 |
| 171.40 | 0.0001 |
| 171.25 | 0.0001 |

| | |
|---|---|
| 170.92 | 0.0114 |
| 170.47 | 0.0018 |
| 170.34 | 0.0110 |
| 168.58 | 0.0050 |
| 168.14 | 0.0053 |
| 167.37 | 0.0022 |
| 167.07 | 0.0042 |
| 166.73 | 0.0023 |
| 166.45 | 0.0019 |
| 165.41 | 0.0002 |
| 165.40 | 0.0053 |
| 164.82 | 0.0085 |
| 163.80 | 0.0096 |
| 162.03 | 0.0012 |
| 161.94 | 0.0026 |
| 161.03 | 0.0119 |
| 160.94 | 0.0028 |
| 160.03 | 0.0097 |
| 159.22 | 0.0563 |
| 158.81 | 0.0898 |
| 158.71 | 0.0599 |
| 158.65 | 0.0016 |
| 156.57 | 0.0286 |
| 156.54 | 0.0143 |
| 155.71 | 0.0071 |
| 155.18 | 0.0005 |
| 154.81 | 0.0023 |
| 154.64 | 0.0003 |
| 154.42 | 0.0008 |
| 153.34 | 0.0079 |
| 153.11 | 0.0275 |
| 151.57 | 0.0230 |
| 151.53 | 0.1538 |
| 150.65 | 0.0003 |
| 148.04 | 0.0024 |
| 147.50 | 0.0026 |
| 147.35 | 0.0480 |
| 147.20 | 0.1136 |
| 146.40 | 0.0177 |
| 146.09 | 0.0026 |
| 145.94 | 0.0008 |
| 145.75 | 0.0007 |
| 144.19 | 0.0119 |

Fe$^{+}$(benzene)
B3LYP
m = 4

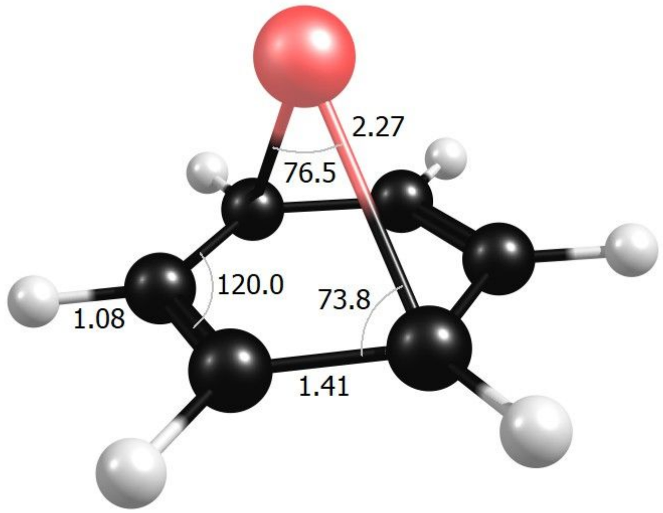


Coordinates:

| | | | |
|---|---|---|---|
| Fe | -0.000604000 | -0.000084000 | 1.125857000 |
| C | 0.000428000 | -1.406299000 | -0.659285000 |
| C | 1.222309000 | -0.700001000 | -0.712503000 |
| C | -1.221471000 | -0.700128000 | -0.713789000 |
| H | 2.158439000 | -1.241925000 | -0.735064000 |
| H | -2.157522000 | -1.242148000 | -0.737326000 |
| C | 1.222236000 | 0.700231000 | -0.712399000 |
| C | -1.221543000 | 0.700105000 | -0.713688000 |
| H | 2.158308000 | 1.242260000 | -0.734872000 |
| H | -2.157648000 | 1.242036000 | -0.737139000 |
| C | 0.000280000 | 1.406400000 | -0.659085000 |
| H | 0.000218000 | 2.487706000 | -0.651509000 |
| H | 0.000479000 | -2.487607000 | -0.651888000 |

Electronic Transitions

| Wavelength, nm | Oscillator strength |
|---|---|
| 4184.05 | 0.0000 |
| 2001.04 | 0.0000 |
| 1416.31 | 0.0004 |
| 1342.62 | 0.0005 |
| 679.63 | 0.0007 |
| 668.49 | 0.0007 |
| 668.35 | 0.0004 |
| 590.29 | 0.0000 |
| 432.71 | 0.0053 |
| 415.69 | 0.0035 |

| | |
|---|---|
| 413.21 | 0.0011 |
| 383.14 | 0.0000 |
| 374.25 | 0.0000 |
| 342.38 | 0.0099 |
| 318.20 | 0.0000 |
| 317.26 | 0.0007 |
| 315.56 | 0.0000 |
| 307.04 | 0.0000 |
| 285.39 | 0.0022 |
| 280.53 | 0.0039 |
| 272.11 | 0.0010 |
| 252.56 | 0.0000 |
| 249.26 | 0.0000 |
| 247.19 | 0.0383 |
| 241.88 | 0.0317 |
| 238.67 | 0.0000 |
| 230.95 | 0.0009 |
| 229.61 | 0.0006 |
| 229.02 | 0.0051 |
| 225.10 | 0.0001 |
| 223.63 | 0.0024 |
| 210.97 | 0.0000 |
| 208.20 | 0.0140 |
| 203.42 | 0.0003 |
| 200.41 | 0.0142 |
| 197.73 | 0.0000 |
| 197.50 | 0.0000 |
| 195.91 | 0.0071 |
| 195.86 | 0.0000 |
| 195.55 | 0.0012 |
| 193.82 | 0.0055 |
| 193.50 | 0.0000 |
| 191.84 | 0.0008 |
| 189.83 | 0.0069 |
| 189.00 | 0.0020 |
| 188.36 | 0.0000 |
| 186.39 | 0.0062 |
| 186.00 | 0.0000 |
| 185.24 | 0.0000 |
| 182.96 | 0.0348 |
| 182.24 | 0.0004 |
| 177.44 | 0.0217 |
| 172.86 | 0.0000 |
| 171.74 | 0.0000 |
| 171.69 | 0.0000 |
| 170.78 | 0.0195 |

| | |
|---|---|
| 169.49 | 0.0006 |
| 168.12 | 0.0000 |
| 166.18 | 0.0005 |
| 165.97 | 0.0025 |
| 165.72 | 0.1117 |
| 165.35 | 0.0000 |
| 165.12 | 0.0001 |
| 164.79 | 0.0000 |
| 164.76 | 0.0009 |
| 164.75 | 0.0005 |
| 164.22 | 0.0028 |
| 164.11 | 0.0000 |
| 163.07 | 0.0004 |
| 161.87 | 0.0000 |
| 159.11 | 0.0027 |
| 159.08 | 0.0000 |
| 158.82 | 0.0269 |
| 157.79 | 0.0011 |
| 157.78 | 0.0041 |
| 157.59 | 0.0108 |
| 157.14 | 0.0011 |
| 156.19 | 0.0867 |
| 155.32 | 0.1507 |
| 154.77 | 0.0003 |
| 154.57 | 0.0124 |
| 154.13 | 0.0108 |
| 153.49 | 0.1455 |
| 153.17 | 0.0090 |
| 152.26 | 0.0002 |
| 151.65 | 0.0009 |
| 151.64 | 0.0362 |
| 151.48 | 0.0149 |
| 151.13 | 0.0000 |
| 150.51 | 0.0623 |
| 150.14 | 0.1176 |
| 149.82 | 0.0089 |
| 149.35 | 0.0000 |
| 148.44 | 0.0000 |
| 145.33 | 0.0000 |
| 145.00 | 0.0000 |
| 144.87 | 0.0002 |
| 143.79 | 0.0558 |
| 143.49 | 0.1664 |
| 142.61 | 0.0260 |

$Fe^{+}$(benzene)
B3LYP
m = 6

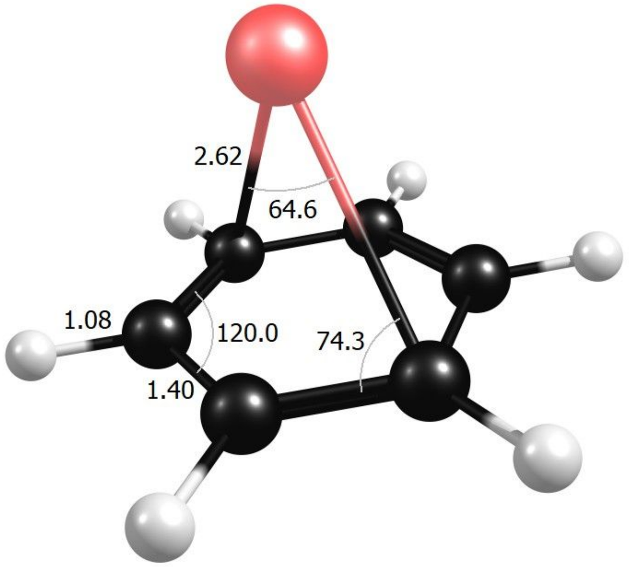


Coordinates:

| | | | |
|---|---|---|---|
| Fe | -1.364064000 | 0.000469000 | -0.000130000 |
| C | 0.844387000 | -0.985933000 | -0.995163000 |
| C | 0.840066000 | 0.369907000 | -1.350382000 |
| C | 0.850115000 | -1.355662000 | 0.354968000 |
| H | 0.831838000 | 0.655481000 | -2.394016000 |
| H | 0.848924000 | -2.402350000 | 0.629560000 |
| C | 0.851567000 | 1.355074000 | -0.354805000 |
| C | 0.839322000 | -0.370482000 | 1.350543000 |
| H | 0.851510000 | 2.401762000 | -0.629397000 |
| H | 0.830517000 | -0.656047000 | 2.394175000 |
| C | 0.845094000 | 0.985354000 | 0.995321000 |
| H | 0.840419000 | 1.746039000 | 1.764864000 |
| H | 0.839159000 | -1.746618000 | -1.764703000 |

Electronic Transitions

| Wavelength, nm | Oscillator strength |
|---|---|
| 6149.52 | 0.0000 |
| 4228.90 | 0.0000 |
| 2248.30 | 0.0000 |
| 2224.28 | 0.0000 |
| 439.87 | 0.0001 |
| 439.19 | 0.0000 |
| 367.20 | 0.0002 |
| 367.05 | 0.0002 |
| 361.03 | 0.0000 |
| 326.58 | 0.0000 |

| | |
|---|---|
| 325.64 | 0.0000 |
| 321.76 | 0.0000 |
| 312.63 | 0.0002 |
| 293.69 | 0.0001 |
| 285.17 | 0.0344 |
| 272.58 | 0.0006 |
| 257.86 | 0.0661 |
| 257.41 | 0.0672 |
| 247.05 | 0.0455 |
| 245.49 | 0.0467 |
| 236.84 | 0.0001 |
| 234.97 | 0.0022 |
| 231.80 | 0.0016 |
| 226.36 | 0.0000 |
| 226.17 | 0.0000 |
| 226.01 | 0.0000 |
| 224.16 | 0.0026 |
| 211.31 | 0.0000 |
| 211.08 | 0.0000 |
| 211.03 | 0.0000 |
| 210.84 | 0.0002 |
| 208.43 | 0.2665 |
| 206.48 | 0.0055 |
| 206.06 | 0.1468 |
| 199.84 | 0.0009 |
| 199.49 | 0.0000 |
| 192.36 | 0.0033 |
| 186.83 | 0.0000 |
| 177.85 | 0.0000 |
| 177.70 | 0.0000 |
| 177.43 | 0.0450 |
| 177.32 | 0.0463 |
| 173.77 | 0.0000 |
| 173.30 | 0.0008 |
| 172.39 | 0.0000 |
| 171.26 | 0.0007 |
| 171.10 | 0.0000 |
| 170.81 | 0.0055 |
| 170.73 | 0.0060 |
| 168.81 | 0.0000 |
| 168.10 | 0.3208 |
| 167.97 | 0.3353 |
| 165.68 | 0.0000 |
| 164.97 | 0.0029 |
| 162.93 | 0.0207 |
| 162.65 | 0.0191 |

| | |
|---|---|
| 162.23 | 0.0000 |
| 161.85 | 0.0000 |
| 161.65 | 0.0001 |
| 161.24 | 0.0001 |
| 161.02 | 0.0000 |
| 160.72 | 0.0748 |
| 159.98 | 0.1182 |
| 159.78 | 0.1136 |
| 159.71 | 0.0045 |
| 159.57 | 0.0000 |
| 158.22 | 0.0090 |
| 158.09 | 0.0000 |
| 155.07 | 0.0001 |
| 154.92 | 0.0000 |
| 154.77 | 0.0001 |
| 154.06 | 0.0001 |
| 154.02 | 0.0002 |
| 153.71 | 0.0005 |
| 149.69 | 0.0003 |
| 149.09 | 0.0015 |
| 148.39 | 0.0012 |
| 148.34 | 0.0001 |
| 145.42 | 0.0000 |
| 145.10 | 0.1592 |
| 145.02 | 0.0000 |
| 144.72 | 0.0072 |
| 144.57 | 0.0050 |
| 143.38 | 0.0000 |
| 141.93 | 0.0000 |
| 141.63 | 0.0001 |
| 141.56 | 0.0496 |
| 141.55 | 0.0218 |
| 141.54 | 0.0006 |
| 141.42 | 0.0182 |
| 140.99 | 0.0000 |
| 138.63 | 0.0081 |
| 138.52 | 0.0049 |
| 138.45 | 0.0000 |
| 138.44 | 0.0031 |
| 138.36 | 0.0000 |
| 137.85 | 0.0150 |
| 137.23 | 0.0032 |
| 137.14 | 0.0062 |
| 137.03 | 0.0000 |

$Fe^+$(benzene)
M06-L
m = 2

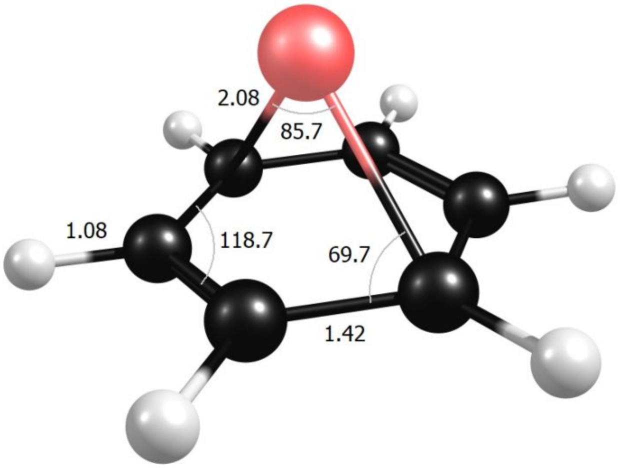


Coordinates:

| | | | |
|---|---|---|---|
| Fe | 0.000020000 | -0.035586000 | 0.943765000 |
| C | 1.426799000 | 0.025707000 | -0.635821000 |
| C | 0.712186000 | -1.189316000 | -0.602036000 |
| C | 0.713670000 | 1.229999000 | -0.539053000 |
| H | 1.241764000 | -2.131600000 | -0.551511000 |
| H | 1.243948000 | 2.167350000 | -0.434220000 |
| C | -0.712372000 | -1.189226000 | -0.602011000 |
| C | -0.713532000 | 1.230086000 | -0.539022000 |
| H | -1.242067000 | -2.131444000 | -0.551487000 |
| H | -1.243690000 | 2.167504000 | -0.434186000 |
| C | -1.426825000 | 0.025888000 | -0.635777000 |
| H | -2.508372000 | 0.027474000 | -0.622045000 |
| H | 2.508347000 | 0.027141000 | -0.622126000 |

Electronic Transitions

| Wavelength, nm | Oscillator strength |
|---|---|
| 2530.58 | 0.0000 |
| 1534.89 | 0.0000 |
| 1327.88 | 0.0002 |
| 832.62 | 0.0000 |
| 794.92 | 0.0010 |
| 737.92 | 0.0012 |
| 736.59 | 0.0018 |
| 700.24 | 0.0003 |
| 516.42 | 0.0023 |
| 470.43 | 0.0005 |
| 426.57 | 0.0067 |
| 423.95 | 0.0057 |
| 369.51 | 0.0008 |

| | |
|---|---|
| 367.42 | 0.0004 |
| 350.42 | 0.0035 |
| 341.15 | 0.0010 |
| 308.51 | 0.0016 |
| 307.26 | 0.006 |
| 305.47 | 0.0011 |
| 301.27 | 0.0009 |
| 300.04 | 0.0023 |
| 290.58 | 0.0037 |
| 289.69 | 0.0017 |
| 285.45 | 0.0050 |
| 279.88 | 0.0028 |
| 277.36 | 0.0005 |
| 277.09 | 0.0049 |
| 275.23 | 0.0028 |
| 263.59 | 0.0032 |
| 262.39 | 0.0033 |
| 258.99 | 0.0019 |
| 251.84 | 0.0016 |
| 249.37 | 0.0016 |
| 247.66 | 0.0007 |
| 244.06 | 0.0002 |
| 238.97 | 0.0001 |
| 238.24 | 0.0010 |
| 234.15 | 0.0000 |
| 226.06 | 0.0013 |
| 224.41 | 0.0017 |
| 220.30 | 0.0002 |
| 212.56 | 0.0001 |
| 205.67 | 0.0003 |
| 204.20 | 0.0004 |
| 202.75 | 0.0007 |
| 201.61 | 0.0007 |
| 199.87 | 0.0023 |
| 194.82 | 0.0029 |
| 187.47 | 0.0032 |
| 186.48 | 0.0003 |
| 185.07 | 0.0045 |
| 184.96 | 0.0000 |
| 183.89 | 0.0001 |
| 180.20 | 0.0011 |
| 179.39 | 0.0008 |
| 177.72 | 0.0000 |
| 177.47 | 0.0019 |
| 176.52 | 0.0001 |
| 176.52 | 0.0000 |

| | |
|---|---|
| 175.88 | 0.0009 |
| 175.44 | 0.0006 |
| 174.76 | 0.0013 |
| 170.1 | 0.0002 |
| 170.08 | 0.0077 |
| 169.14 | 0.0055 |
| 168.52 | 0.0005 |
| 168.05 | 0.0012 |
| 167.66 | 0.0099 |
| 165.74 | 0.0048 |
| 164.23 | 0.0001 |
| 162.22 | 0.0054 |
| 162.08 | 0.0021 |
| 161.4 | 0.0013 |
| 160.99 | 0.0003 |
| 160.81 | 0.0013 |
| 160.67 | 0.0000 |
| 160.29 | 0.0133 |
| 160.11 | 0.0004 |
| 159.71 | 0.0001 |
| 159.06 | 0.0047 |
| 158.26 | 0.0032 |
| 158.16 | 0.0045 |
| 157.97 | 0.0000 |
| 157.51 | 0.006 |
| 157.24 | 0.039 |
| 157.09 | 0.0017 |
| 156.65 | 0.0344 |
| 156.57 | 0.0005 |
| 155.29 | 0.0000 |
| 155.19 | 0.0026 |
| 155.08 | 0.0000 |
| 153.57 | 0.0164 |
| 153.39 | 0.0339 |
| 153.26 | 0.0001 |
| 151.01 | 0.1068 |
| 150.9 | 0.0017 |
| 149.77 | 0.0027 |
| 148.99 | 0.0152 |
| 148.25 | 0.0000 |
| 147.6 | 0.0099 |

$Fe^+$(benzene)
M06-L
m = 4

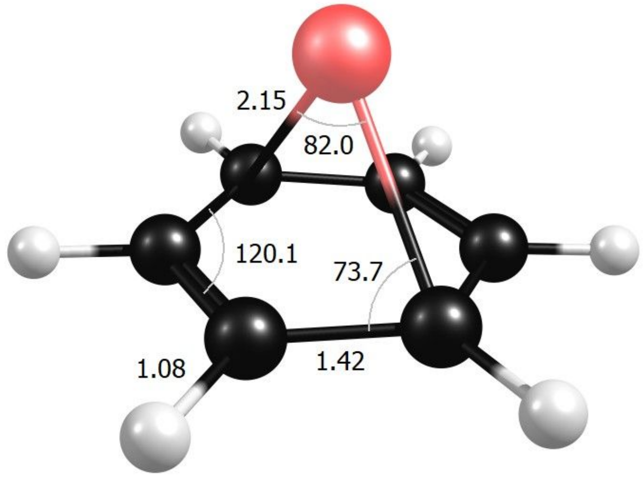


Coordinates:

| | | | |
|---|---|---|---|
| Fe | 0.000000000 | 0.000000000 | 1.038606000 |
| C | 0.000001000 | -1.408379000 | -0.581806000 |
| C | 1.223463000 | -0.697926000 | -0.671286000 |
| C | -1.223463000 | -0.697927000 | -0.671283000 |
| H | 2.159865000 | -1.239846000 | -0.697166000 |
| H | -2.159864000 | -1.239849000 | -0.697163000 |
| C | 1.223463000 | 0.697927000 | -0.671282000 |
| C | -1.223463000 | 0.697926000 | -0.671286000 |
| H | 2.159864000 | 1.239849000 | -0.697162000 |
| H | -2.159865000 | 1.239846000 | -0.697166000 |
| C | -0.000001000 | 1.408379000 | -0.581805000 |
| H | -0.000002000 | 2.489345000 | -0.561306000 |
| H | 0.000002000 | -2.489345000 | -0.561307000 |

Electronic Transitions

| Wavelength, nm | Oscillator strength |
|---|---|
| 2193.55 | 0.0000 |
| 1252.40 | 0.0010 |
| 1165.71 | 0.0005 |
| 1076.25 | 0.0006 |
| 630.07 | 0.0000 |
| 557.70 | 0.0016 |
| 547.21 | 0.0014 |
| 406.14 | 0.0095 |
| 405.40 | 0.0000 |
| 383.23 | 0.0043 |
| 366.44 | 0.0000 |

| | |
|---|---|
| 350.22 | 0.0001 |
| 336.85 | 0.0015 |
| 330.76 | 0.0097 |
| 317.38 | 0.0000 |
| 314.18 | 0.0000 |
| 300.12 | 0.0018 |
| 297.82 | 0.0007 |
| 293.64 | 0.0009 |
| 287.58 | 0.0020 |
| 273.48 | 0.0008 |
| 268.28 | 0.0206 |
| 266.42 | 0.0353 |
| 258.53 | 0.0000 |
| 256.87 | 0.0002 |
| 249.65 | 0.0000 |
| 245.23 | 0.0000 |
| 242.46 | 0.0000 |
| 241.95 | 0.0000 |
| 238.92 | 0.0000 |
| 238.63 | 0.0000 |
| 238.08 | 0.0000 |
| 234.82 | 0.0000 |
| 231.83 | 0.0000 |
| 231.39 | 0.0000 |
| 222.23 | 0.0007 |
| 216.76 | 0.0015 |
| 212.11 | 0.0034 |
| 211.48 | 0.0006 |
| 211.39 | 0.0000 |
| 209.72 | 0.0004 |
| 209.40 | 0.0009 |
| 202.36 | 0.0005 |
| 199.46 | 0.0009 |
| 197.21 | 0.0000 |
| 196.69 | 0.0011 |
| 194.40 | 0.0005 |
| 194.17 | 0.0007 |
| 193.28 | 0.0004 |
| 191.05 | 0.0002 |
| 190.30 | 0.0000 |
| 188.51 | 0.0037 |
| 186.85 | 0.0145 |
| 185.99 | 0.0067 |
| 184.63 | 0.0000 |
| 184.48 | 0.0025 |
| 184.15 | 0.0000 |

| | |
|---|---|
| 183.35 | 0.0002 |
| 179.23 | 0.0041 |
| 174.03 | 0.0025 |
| 171.56 | 0.0035 |
| 169.57 | 0.0000 |
| 169.26 | 0.0008 |
| 169.00 | 0.0001 |
| 168.83 | 0.0032 |
| 168.05 | 0.0000 |
| 167.56 | 0.0017 |
| 166.78 | 0.0002 |
| 166.68 | 0.0000 |
| 166.10 | 0.0000 |
| 166.01 | 0.0001 |
| 164.64 | 0.0001 |
| 164.14 | 0.0022 |
| 163.07 | 0.0000 |
| 162.50 | 0.0814 |
| 161.16 | 0.0000 |
| 160.25 | 0.0000 |
| 159.50 | 0.0001 |
| 159.29 | 0.0000 |
| 157.97 | 0.0004 |
| 157.92 | 0.0100 |
| 157.48 | 0.0491 |
| 157.47 | 0.0130 |
| 156.84 | 0.0000 |
| 156.79 | 0.0406 |
| 156.18 | 0.1877 |
| 155.95 | 0.0006 |
| 152.95 | 0.0412 |
| 152.75 | 0.0006 |
| 152.30 | 0.0845 |
| 152.16 | 0.0111 |
| 151.51 | 0.0002 |
| 151.29 | 0.0000 |
| 150.93 | 0.0005 |
| 150.19 | 0.0152 |
| 149.12 | 0.1372 |
| 148.96 | 0.0000 |
| 148.87 | 0.0495 |
| 147.89 | 0.0000 |

$Fe^+$(benzene)
M06-L
m = 6

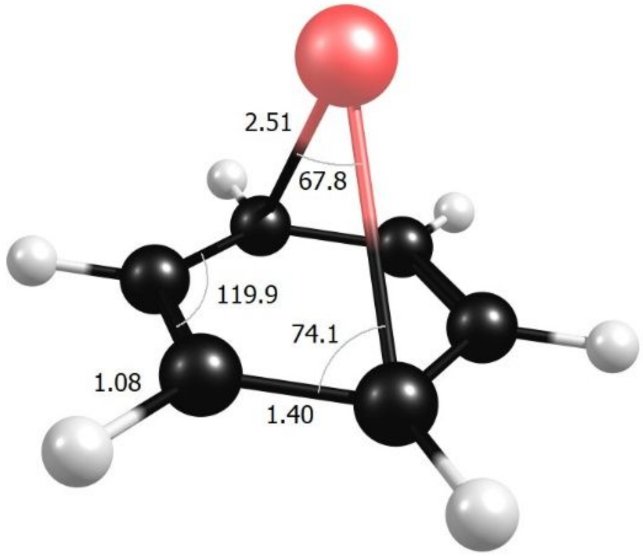


Coordinates:

| | | | |
|---|---|---|---|
| Fe | -1.291683000 | -0.000003000 | 0.000017000 |
| C | 0.797478000 | 0.858519000 | -1.104590000 |
| C | 0.818367000 | 1.387608000 | 0.188434000 |
| C | 0.789025000 | -0.530907000 | -1.293662000 |
| H | 0.815918000 | 2.459605000 | 0.334857000 |
| H | 0.766041000 | -0.939507000 | -2.295011000 |
| C | 0.789065000 | 0.530911000 | 1.293641000 |
| C | 0.818369000 | -1.387604000 | -0.188455000 |
| H | 0.766113000 | 0.939509000 | 2.294992000 |
| H | 0.815922000 | -2.459601000 | -0.334879000 |
| C | 0.797519000 | -0.858515000 | 1.104569000 |
| H | 0.780457000 | -1.519971000 | 1.960417000 |
| H | 0.780377000 | 1.519976000 | -1.960436000 |

Calculations failed to predict electronic transitions.

$Fe^{+}$(benzene)
MN15-L
m = 2

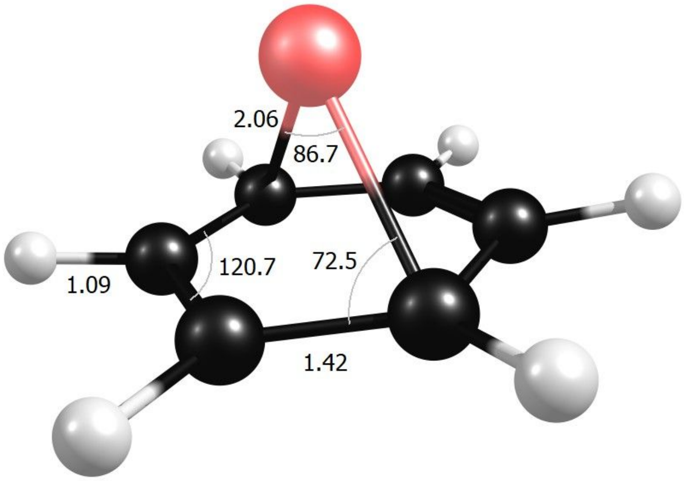


Coordinates:

| | | | |
|---|---|---|---|
| Fe | -0.000001000 | 0.008413000 | 0.928566000 |
| C | 0.718191000 | 1.212561000 | -0.576253000 |
| C | 1.444193000 | -0.004277000 | -0.622356000 |
| C | -0.719080000 | 1.212066000 | -0.576289000 |
| H | 2.536383000 | -0.003339000 | -0.586994000 |
| H | -1.251853000 | 2.162323000 | -0.474817000 |
| C | 0.719241000 | -1.223081000 | -0.561556000 |
| C | -1.444192000 | -0.005286000 | -0.622352000 |
| H | 1.250163000 | -2.172672000 | -0.448698000 |
| H | -2.536382000 | -0.005165000 | -0.586974000 |
| C | -0.718349000 | -1.223576000 | -0.561520000 |
| H | -1.248596000 | -2.173535000 | -0.448579000 |
| H | 1.250284000 | 2.163192000 | -0.474707000 |

Electronic Transitions

| Wavelength, nm | Oscillator strength |
|---|---|
| 2774.66 | 0.0000 |
| 1291.23 | 0.0000 |
| 1125.35 | 0.0004 |
| 925.49 | 0.0002 |
| 905.58 | 0.0001 |
| 893.33 | 0.0002 |
| 714.54 | 0.0025 |
| 704.58 | 0.0018 |
| 526.51 | 0.0037 |
| 464.28 | 0.0016 |
| 436.34 | 0.0064 |

| | |
|---|---|
| 423.88 | 0.0085 |
| 387.72 | 0.0000 |
| 376.82 | 0.0002 |
| 361.89 | 0.0008 |
| 323.92 | 0.0003 |
| 311.89 | 0.0005 |
| 297.60 | 0.0003 |
| 296.44 | 0.0009 |
| 293.79 | 0.0000 |
| 284.99 | 0.0027 |
| 281.15 | 0.0091 |
| 280.90 | 0.0104 |
| 273.43 | 0.0110 |
| 273.07 | 0.0001 |
| 264.27 | 0.0052 |
| 262.14 | 0.0008 |
| 259.60 | 0.0016 |
| 257.32 | 0.0013 |
| 255.43 | 0.0031 |
| 253.77 | 0.0018 |
| 240.26 | 0.0005 |
| 235.31 | 0.0017 |
| 231.48 | 0.0003 |
| 227.53 | 0.0000 |
| 223.43 | 0.0002 |
| 212.21 | 0.0007 |
| 211.11 | 0.0001 |
| 209.20 | 0.0017 |
| 206.97 | 0.0007 |
| 203.17 | 0.0000 |
| 202.48 | 0.0067 |
| 200.92 | 0.0044 |
| 195.44 | 0.0000 |
| 192.90 | 0.0015 |
| 191.48 | 0.0007 |
| 190.64 | 0.0018 |
| 190.36 | 0.0015 |
| 188.63 | 0.0022 |
| 183.63 | 0.0000 |
| 181.66 | 0.0075 |
| 181.36 | 0.0016 |
| 179.22 | 0.0011 |
| 178.21 | 0.0003 |
| 177.93 | 0.0039 |
| 173.18 | 0.0063 |
| 172.56 | 0.0001 |

| | |
|---|---|
| 172.02 | 0.0000 |
| 170.61 | 0.0466 |
| 170.38 | 0.0023 |
| 169.72 | 0.0746 |
| 168.26 | 0.0036 |
| 168.06 | 0.0000 |
| 167.06 | 0.0002 |
| 166.09 | 0.0006 |
| 165.38 | 0.0000 |
| 164.81 | 0.0030 |
| 164.69 | 0.0001 |
| 163.93 | 0.0003 |
| 163.36 | 0.0006 |
| 162.16 | 0.0003 |
| 161.31 | 0.0001 |
| 160.89 | 0.0033 |
| 160.60 | 0.0005 |
| 159.76 | 0.0015 |
| 159.32 | 0.0055 |
| 158.57 | 0.0047 |
| 156.34 | 0.0001 |
| 155.93 | 0.0000 |
| 155.52 | 0.0000 |
| 154.90 | 0.0039 |
| 152.89 | 0.0003 |
| 152.30 | 0.0004 |
| 152.27 | 0.0007 |
| 151.77 | 0.0014 |
| 151.26 | 0.0000 |
| 150.81 | 0.0001 |
| 150.26 | 0.0004 |
| 150.17 | 0.0078 |
| 150.12 | 0.0005 |
| 149.10 | 0.0010 |
| 148.91 | 0.0006 |
| 148.38 | 0.0268 |
| 147.53 | 0.0161 |
| 147.42 | 0.0021 |
| 146.68 | 0.0006 |
| 146.36 | 0.0142 |
| 146.21 | 0.0053 |
| 146.04 | 0.1717 |
| 145.84 | 0.0029 |

$Fe^{+}$(benzene)
MN15-L
m = 4

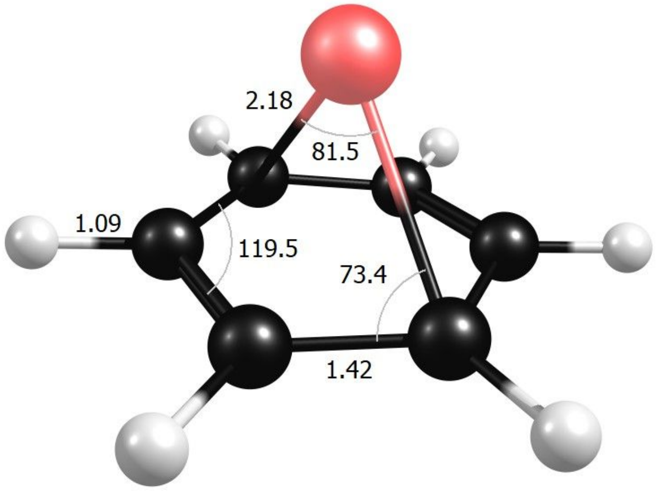


Coordinates:

| | | | |
|---|---|---|---|
| Fe | -0.000001000 | 0.000000000 | 1.038641000 |
| C | 0.000482000 | 1.414425000 | -0.697039000 |
| C | -1.231910000 | 0.717474000 | -0.616759000 |
| C | 1.232399000 | 0.716637000 | -0.616761000 |
| H | -2.178216000 | 1.262989000 | -0.613267000 |
| H | 2.179078000 | 1.261506000 | -0.613271000 |
| C | -1.232398000 | -0.716638000 | -0.616762000 |
| C | 1.231911000 | -0.717475000 | -0.616757000 |
| H | -2.179077000 | -1.261506000 | -0.613273000 |
| H | 2.178217000 | -1.262990000 | -0.613263000 |
| C | -0.000481000 | -1.414425000 | -0.697038000 |
| H | -0.000854000 | -2.508971000 | -0.692444000 |
| H | 0.000855000 | 2.508971000 | -0.692446000 |

Electronic Transitions

| Wavelength, nm | Oscillator strength |
|---|---|
| 2075.76 | 0.0000 |
| 1389.33 | 0.0006 |
| 1041.73 | 0.0002 |
| 901.81 | 0.0009 |
| 754.66 | 0.0042 |
| 691.93 | 0.0000 |
| 522.77 | 0.0000 |
| 488.24 | 0.0029 |
| 407.70 | 0.0073 |
| 364.27 | 0.0000 |
| 343.79 | 0.0001 |

| | |
|---|---|
| 340.39 | 0.0052 |
| 314.00 | 0.0000 |
| 298.05 | 0.0117 |
| 288.96 | 0.0054 |
| 286.22 | 0.0060 |
| 277.16 | 0.0002 |
| 271.02 | 0.0036 |
| 257.77 | 0.0000 |
| 254.54 | 0.0089 |
| 234.69 | 0.0267 |
| 231.41 | 0.0001 |
| 230.75 | 0.0423 |
| 228.32 | 0.0000 |
| 227.03 | 0.0021 |
| 216.92 | 0.0073 |
| 214.82 | 0.0011 |
| 211.93 | 0.0000 |
| 209.76 | 0.0005 |
| 208.11 | 0.0002 |
| 208.04 | 0.0000 |
| 207.15 | 0.0000 |
| 206.14 | 0.0000 |
| 204.72 | 0.0103 |
| 202.92 | 0.0000 |
| 201.67 | 0.0018 |
| 201.61 | 0.0059 |
| 201.31 | 0.0012 |
| 198.04 | 0.0000 |
| 195.07 | 0.0135 |
| 192.74 | 0.0000 |
| 189.66 | 0.0000 |
| 188.70 | 0.0000 |
| 186.85 | 0.0000 |
| 185.77 | 0.0020 |
| 184.06 | 0.0026 |
| 181.99 | 0.0004 |
| 181.78 | 0.0073 |
| 180.43 | 0.0115 |
| 178.50 | 0.0115 |
| 177.82 | 0.0000 |
| 173.40 | 0.0000 |
| 173.00 | 0.0008 |
| 171.78 | 0.0046 |
| 170.15 | 0.0000 |
| 169.94 | 0.0064 |
| 169.60 | 0.0028 |

| | |
|---|---|
| 169.30 | 0.0000 |
| 169.06 | 0.0051 |
| 168.92 | 0.0033 |
| 166.58 | 0.0001 |
| 166.43 | 0.0020 |
| 165.70 | 0.0037 |
| 165.66 | 0.0000 |
| 164.31 | 0.0013 |
| 164.23 | 0.0000 |
| 163.86 | 0.0030 |
| 162.41 | 0.0147 |
| 161.94 | 0.0021 |
| 160.38 | 0.0039 |
| 160.27 | 0.0359 |
| 159.35 | 0.0000 |
| 158.93 | 0.0064 |
| 158.73 | 0.0036 |
| 158.07 | 0.0000 |
| 157.30 | 0.0033 |
| 156.39 | 0.0000 |
| 155.92 | 0.0141 |
| 155.01 | 0.0002 |
| 154.80 | 0.1297 |
| 152.32 | 0.0532 |
| 151.46 | 0.0119 |
| 151.36 | 0.0000 |
| 151.06 | 0.0052 |
| 150.80 | 0.0000 |
| 150.36 | 0.0011 |
| 148.19 | 0.0073 |
| 147.81 | 0.0000 |
| 146.91 | 0.0056 |
| 146.64 | 0.2818 |
| 146.15 | 0.2887 |
| 144.26 | 0.0000 |
| 143.65 | 0.0032 |
| 141.83 | 0.0141 |
| 141.79 | 0.0000 |
| 141.22 | 0.0074 |
| 141.00 | 0.0034 |
| 140.60 | 0.0026 |
| 140.48 | 0.0233 |
| 140.06 | 0.1109 |

$Fe^{+}$(benzene)
MN15-L
m = 6

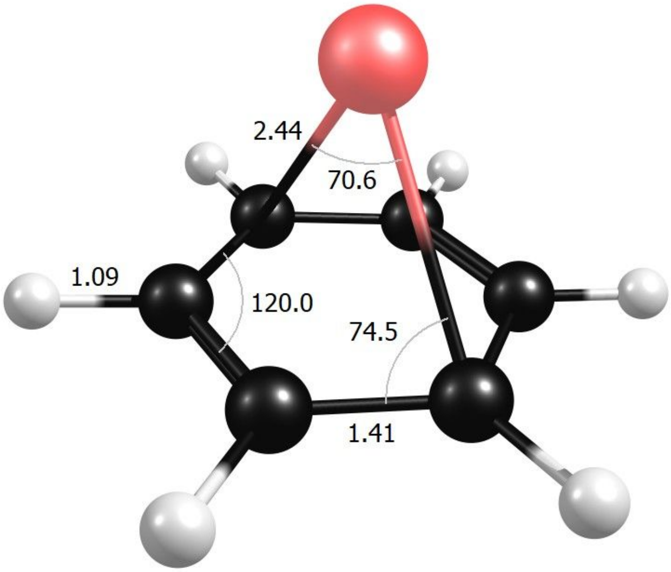


Coordinates:

| | | | |
|---|---|---|---|
| Fe | -1.244345000 | 0.000000000 | 0.000000000 |
| C | 0.783992000 | 1.191968000 | 0.759470000 |
| C | 0.750787000 | -0.064770000 | 1.409989000 |
| C | 0.786876000 | 1.256379000 | -0.647862000 |
| H | 0.710009000 | -0.114632000 | 2.501279000 |
| H | 0.770705000 | 2.226912000 | -1.151493000 |
| C | 0.786876000 | -1.256379000 | 0.647862000 |
| C | 0.750788000 | 0.064770000 | -1.409988000 |
| H | 0.770704000 | -2.226912000 | 1.151493000 |
| H | 0.710009000 | 0.114632000 | -2.501279000 |
| C | 0.783992000 | -1.191968000 | -0.759470000 |
| H | 0.765837000 | -2.112284000 | -1.349783000 |
| H | 0.765837000 | 2.112284000 | 1.349783000 |

Electronic Transitions

| Wavelength, nm | Oscillator strength |
|---|---|
| 3660.90 | 0.0000 |
| 1222.25 | 0.0000 |
| 1015.67 | 0.0002 |
| 999.41 | 0.0002 |
| 521.47 | 0.0010 |
| 521.20 | 0.0000 |
| 366.64 | 0.0002 |
| 317.64 | 0.0001 |
| 317.26 | 0.0000 |

| | |
|---|---|
| 316.60 | 0.0014 |
| 315.00 | 0.0000 |
| 288.26 | 0.0000 |
| 286.65 | 0.0000 |
| 281.50 | 0.0001 |
| 272.24 | 0.0000 |
| 269.47 | 0.0007 |
| 248.79 | 0.0653 |
| 248.53 | 0.0581 |
| 245.86 | 0.0764 |
| 244.99 | 0.0543 |
| 230.16 | 0.0000 |
| 229.12 | 0.0001 |
| 223.52 | 0.0275 |
| 221.10 | 0.0020 |
| 220.81 | 0.0293 |
| 219.69 | 0.0027 |
| 212.87 | 0.0008 |
| 212.38 | 0.0007 |
| 212.00 | 0.0000 |
| 211.68 | 0.0004 |
| 205.87 | 0.0003 |
| 205.31 | 0.0000 |
| 190.24 | 0.0046 |
| 190.15 | 0.0024 |
| 188.14 | 0.0066 |
| 186.42 | 0.0012 |
| 185.97 | 0.3426 |
| 183.57 | 0.0000 |
| 180.21 | 0.0031 |
| 180.10 | 0.0080 |
| 179.38 | 0.0114 |
| 178.65 | 0.0045 |
| 174.97 | 0.0004 |
| 174.32 | 0.0006 |
| 168.70 | 0.0420 |
| 168.40 | 0.0448 |
| 167.79 | 0.0000 |
| 167.51 | 0.0012 |
| 167.03 | 0.0000 |
| 166.97 | 0.0006 |
| 165.55 | 0.0003 |
| 165.54 | 0.0000 |
| 164.05 | 0.0000 |
| 163.30 | 0.0372 |
| 162.63 | 0.0000 |

| | |
|---|---|
| 162.46 | 0.0490 |
| 161.21 | 0.0034 |
| 161.03 | 0.1861 |
| 159.18 | 0.3702 |
| 157.76 | 0.0000 |
| 157.13 | 0.0265 |
| 156.82 | 0.0257 |
| 155.63 | 0.0001 |
| 155.26 | 0.0002 |
| 155.22 | 0.0000 |
| 154.78 | 0.0006 |
| 154.07 | 0.0000 |
| 153.81 | 0.0000 |
| 153.57 | 0.0009 |
| 153.53 | 0.0028 |
| 153.20 | 0.1691 |
| 152.94 | 0.0080 |
| 152.62 | 0.0000 |
| 151.97 | 0.0000 |
| 151.59 | 0.0355 |
| 151.24 | 0.0000 |
| 150.82 | 0.0034 |
| 150.46 | 0.0025 |
| 149.85 | 0.0027 |
| 149.52 | 0.0059 |
| 147.42 | 0.0000 |
| 146.92 | 0.0000 |
| 146.22 | 0.0125 |
| 144.52 | 0.0597 |
| 144.08 | 0.0974 |
| 143.78 | 0.0000 |
| 143.56 | 0.0000 |
| 142.29 | 0.0001 |
| 141.31 | 0.0000 |
| 140.71 | 0.0004 |
| 140.65 | 0.0000 |
| 140.56 | 0.0008 |
| 139.83 | 0.0001 |
| 139.68 | 0.0005 |
| 139.48 | 0.0000 |
| 138.57 | 0.1053 |
| 138.31 | 0.1098 |
| 137.12 | 0.0257 |
| 136.85 | 0.0000 |
| 136.74 | 0.0004 |

$Fe^{+}(benzene)_2$
B3LYP
m = 2

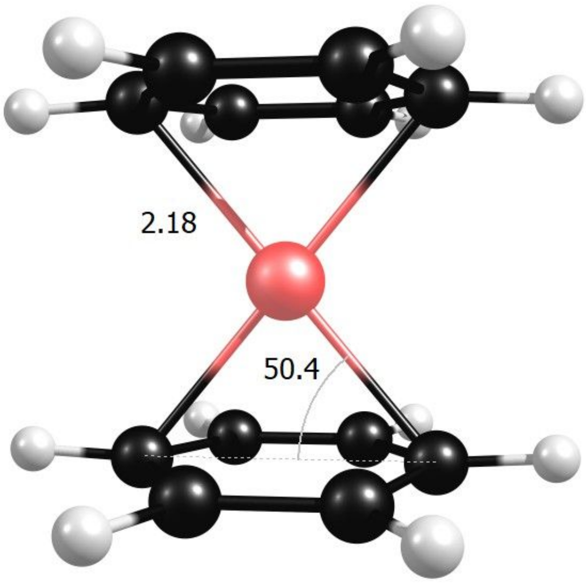


Benzene Dihedral: 6°
Coordinates:

| | | | |
|---|---|---|---|
| Fe | 0.000000000 | 0.000000000 | 0.000000000 |
| C | 0.750398000 | -2.116010000 | 0.006981000 |
| C | 0.093276000 | -1.884217000 | 1.217044000 |
| C | 0.093156000 | -1.814694000 | -1.205699000 |
| H | 0.595055000 | -2.059604000 | 2.158236000 |
| H | 0.621038000 | -1.937945000 | -2.141304000 |
| C | -1.215030000 | -1.353577000 | 1.203124000 |
| C | -1.257432000 | -1.403473000 | -1.219756000 |
| H | -1.703315000 | -1.118642000 | 2.138852000 |
| H | -1.757197000 | -1.222392000 | -2.160941000 |
| C | -1.914653000 | -1.171886000 | -0.009655000 |
| H | -2.932452000 | -0.808168000 | 0.003090000 |
| H | 1.770181000 | -2.474157000 | -0.005750000 |
| C | -0.093156000 | 1.814694000 | 1.205699000 |
| H | -0.621038000 | 1.937945000 | 2.141304000 |
| C | -0.750398000 | 2.116010000 | -0.006981000 |
| H | -1.770181000 | 2.474157000 | 0.005750000 |
| H | -0.595055000 | 2.059604000 | -2.158236000 |
| C | -0.093276000 | 1.884217000 | -1.217044000 |
| C | 1.215030000 | 1.353577000 | -1.203124000 |
| C | 1.257432000 | 1.403473000 | 1.219756000 |
| C | 1.914653000 | 1.171886000 | 0.009655000 |
| H | 1.757197000 | 1.222392000 | 2.160941000 |
| H | 2.932452000 | 0.808168000 | -0.003090000 |
| H | 1.703315000 | 1.118642000 | -2.138852000 |

Electronic Transitions

| Wavelength, nm | Oscillator strength |
|---|---|
| 2828.55 | 0.0000 |
| 1711.47 | 0.0000 |
| 1361.20 | 0.0000 |
| 1025.94 | 0.0000 |
| 688.21 | 0.0000 |
| 648.24 | 0.0000 |
| 599.31 | 0.0000 |
| 596.71 | 0.0000 |
| 474.81 | 0.0000 |
| 443.48 | 0.0000 |
| 397.22 | 0.0000 |
| 396.99 | 0.0000 |
| 384.58 | 0.0000 |
| 377.17 | 0.0124 |
| 373.92 | 0.0000 |
| 372.83 | 0.0099 |
| 372.02 | 0.0000 |
| 344.98 | 0.0000 |
| 329.19 | 0.0000 |
| 324.61 | 0.0102 |
| 308.86 | 0.0000 |
| 308.79 | 0.0000 |
| 301.47 | 0.0288 |
| 299.71 | 0.0000 |
| 299.39 | 0.0000 |
| 297.15 | 0.0000 |
| 288.39 | 0.0000 |
| 276.99 | 0.0000 |
| 267.52 | 0.0205 |
| 265.44 | 0.0000 |
| 264.79 | 0.0000 |
| 263.21 | 0.0000 |
| 260.91 | 0.0000 |
| 260.89 | 0.0000 |
| 250.01 | 0.0000 |
| 248.68 | 0.0249 |
| 241.69 | 0.0000 |
| 237.67 | 0.0000 |
| 235.80 | 0.0000 |
| 235.11 | 0.0242 |
| 233.58 | 0.0000 |
| 230.51 | 0.0000 |
| 229.44 | 0.0025 |
| 227.94 | 0.1424 |

| | |
|---|---|
| 225.49 | 0.0000 |
| 222.09 | 0.0000 |
| 214.76 | 0.0000 |
| 214.63 | 0.0000 |
| 209.80 | 0.0045 |
| 209.70 | 0.0000 |
| 209.67 | 0.0000 |
| 208.88 | 0.0020 |
| 206.97 | 0.0000 |
| 198.88 | 0.0040 |
| 198.04 | 0.0000 |
| 197.99 | 0.0000 |
| 197.97 | 0.0043 |
| 197.42 | 0.2207 |
| 194.65 | 0.0000 |
| 192.79 | 0.0529 |
| 192.10 | 0.0000 |
| 190.64 | 0.0000 |
| 189.32 | 0.0000 |
| 188.60 | 0.0000 |
| 188.49 | 0.0001 |
| 188.42 | 0.0000 |
| 188.22 | 0.0000 |
| 187.31 | 0.0000 |
| 183.17 | 0.0000 |
| 182.42 | 0.0000 |
| 181.76 | 0.0000 |
| 181.22 | 0.0000 |
| 180.74 | 0.0001 |
| 180.38 | 0.0021 |
| 180.30 | 0.0008 |
| 179.76 | 0.0032 |
| 179.75 | 0.0036 |
| 179.14 | 0.0000 |
| 179.07 | 0.0070 |
| 179.00 | 0.0000 |
| 178.84 | 0.0074 |
| 178.5 | 0.0000 |
| 177.93 | 0.0000 |
| 177.43 | 0.0005 |
| 177.21 | 0.0021 |
| 177.08 | 0.0001 |
| 176.35 | 0.0000 |
| 176.33 | 0.0000 |
| 176.15 | 0.2339 |
| 175.42 | 0.0000 |

| | |
|---|---|
| 174.99 | 0.0000 |
| 174.95 | 0.0008 |
| 174.89 | 0.0002 |
| 174.52 | 0.0019 |
| 173.87 | 0.0119 |
| 173.83 | 0.0000 |
| 173.17 | 0.0000 |
| 172.72 | 0.0000 |
| 172.12 | 0.0000 |
| 171.80 | 0.0040 |

$Fe^{+}(benzene)_2$
B3LYP
m = 4

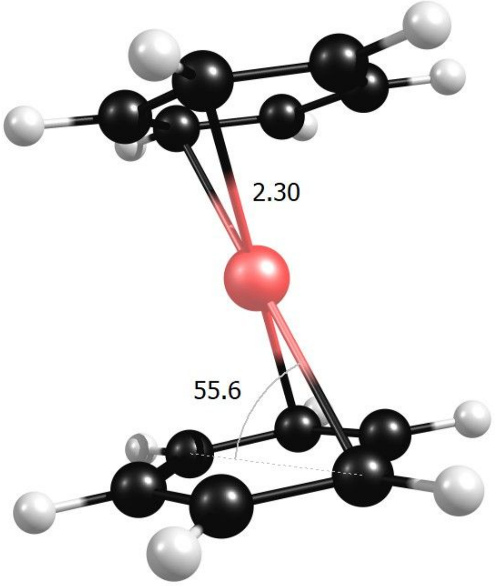


Benzene Dihedral: 2°
Coordinates:

| | | | |
|---|---|---|---|
| Fe | 0.000000000 | 0.000000000 | 0.000000000 |
| C | 0.605957000 | -2.219442000 | 0.052524000 |
| C | -0.101894000 | -2.130446000 | 1.261579000 |
| C | -0.061815000 | -1.982972000 | -1.167508000 |
| H | 0.403395000 | -2.333260000 | 2.196487000 |
| H | 0.463856000 | -2.100673000 | -2.105146000 |
| C | -1.449575000 | -1.767688000 | 1.260409000 |
| C | -1.428110000 | -1.660757000 | -1.161682000 |
| H | -1.986706000 | -1.683668000 | 2.195428000 |
| H | -1.946643000 | -1.500895000 | -2.097659000 |
| C | -2.113498000 | -1.532447000 | 0.047297000 |
| H | -3.162059000 | -1.267214000 | 0.047822000 |
| H | 1.644924000 | -2.519267000 | 0.052861000 |
| C | 0.061815000 | 1.982972000 | 1.167508000 |
| H | -0.463856000 | 2.100673000 | 2.105146000 |
| C | -0.605957000 | 2.219442000 | -0.052524000 |
| H | -1.644924000 | 2.519267000 | -0.052861000 |
| H | -0.403395000 | 2.333260000 | -2.196487000 |
| C | 0.101894000 | 2.130446000 | -1.261579000 |
| C | 1.449575000 | 1.767688000 | -1.260409000 |
| C | 1.428110000 | 1.660757000 | 1.161682000 |
| C | 2.113498000 | 1.532447000 | -0.047297000 |
| H | 1.946643000 | 1.500895000 | 2.097659000 |
| H | 3.162059000 | 1.267214000 | -0.047822000 |
| H | 1.986706000 | 1.683668000 | -2.195428000 |

Electronic Transitions

| Wavelength, nm | Oscillator strength |
|---|---|
| 3750.45 | 0.0000 |
| 2855.05 | 0.0000 |
| 1009.81 | 0.0000 |
| 1006.99 | 0.0000 |
| 565.38 | 0.0000 |
| 554.23 | 0.0000 |
| 425.82 | 0.0000 |
| 414.28 | 0.0000 |
| 390.79 | 0.0001 |
| 373.67 | 0.0000 |
| 372.37 | 0.0681 |
| 370.89 | 0.0005 |
| 358.70 | 0.0000 |
| 353.31 | 0.0000 |
| 346.03 | 0.0002 |
| 336.04 | 0.0000 |
| 327.22 | 0.0000 |
| 319.19 | 0.0000 |
| 314.98 | 0.0000 |
| 312.35 | 0.0002 |
| 311.55 | 0.0154 |
| 309.21 | 0.0491 |
| 290.99 | 0.0000 |
| 279.33 | 0.0000 |
| 275.00 | 0.0000 |
| 271.79 | 0.0000 |
| 267.59 | 0.0000 |
| 266.16 | 0.0000 |
| 264.51 | 0.0004 |
| 261.11 | 0.0003 |
| 257.36 | 0.0164 |
| 254.48 | 0.0381 |
| 251.99 | 0.0000 |
| 247.81 | 0.0000 |
| 245.98 | 0.0000 |
| 239.12 | 0.0000 |
| 238.09 | 0.0412 |
| 237.92 | 0.0006 |
| 233.58 | 0.0000 |
| 229.59 | 0.0140 |
| 228.90 | 0.0000 |
| 221.51 | 0.0000 |
| 219.25 | 0.0000 |
| 218.19 | 0.0185 |

| | |
|---|---|
| 218.07 | 0.0000 |
| 217.63 | 0.0000 |
| 213.06 | 0.0000 |
| 211.99 | 0.0000 |
| 211.52 | 0.0000 |
| 208.67 | 0.0233 |
| 207.11 | 0.0021 |
| 205.66 | 0.0000 |
| 205.12 | 0.0192 |
| 204.95 | 0.0000 |
| 204.42 | 0.0000 |
| 200.06 | 0.0019 |
| 199.31 | 0.0000 |
| 199.09 | 0.0000 |
| 196.34 | 0.0030 |
| 196.22 | 0.0008 |
| 194.84 | 0.0000 |
| 194.44 | 0.0000 |
| 193.51 | 0.0000 |
| 192.58 | 0.0000 |
| 192.25 | 0.0006 |
| 190.80 | 0.0033 |
| 190.77 | 0.0003 |
| 190.49 | 0.0025 |
| 190.06 | 0.0003 |
| 189.56 | 0.0000 |
| 189.15 | 0.0006 |
| 188.56 | 0.0001 |
| 188.25 | 0.0000 |
| 187.72 | 0.0000 |
| 186.84 | 0.0000 |
| 186.24 | 0.0000 |
| 184.80 | 0.0000 |
| 182.15 | 0.0680 |
| 181.15 | 0.0538 |
| 180.84 | 0.0891 |
| 180.30 | 0.0114 |
| 180.29 | 0.0000 |
| 179.44 | 0.0116 |
| 179.23 | 0.0000 |
| 178.99 | 0.0052 |
| 178.73 | 0.0025 |
| 177.45 | 0.0000 |
| 177.08 | 0.0000 |
| 175.81 | 0.0000 |
| 175.60 | 0.0089 |

| | |
|---|---|
| 174.81 | 0.0019 |
| 174.80 | 0.0000 |
| 174.47 | 0.0025 |
| 173.35 | 0.0000 |
| 173.20 | 0.0004 |
| 172.59 | 0.0000 |
| 172.23 | 0.0180 |
| 172.17 | 0.0156 |
| 171.77 | 0.0000 |
| 171.42 | 0.0037 |

$Fe^{+}(benzene)_2$
B3LYP
m = 6

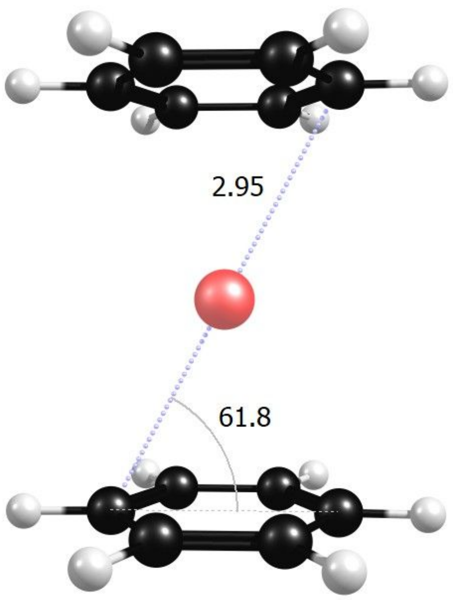


Benzene Dihedral: 0°
Coordinates:

| | | | |
|---|---|---|---|
| Fe | 0.000008000 | -0.000001000 | -0.000002000 |
| C | -2.600522000 | -1.383879000 | 0.186171000 |
| C | -2.598327000 | -0.530746000 | 1.291492000 |
| C | -2.598931000 | -0.853280000 | -1.105256000 |
| H | -2.611377000 | -0.941998000 | 2.292319000 |
| H | -2.612380000 | -1.514393000 | -1.961831000 |
| C | -2.598892000 | 0.853296000 | 1.105255000 |
| C | -2.598339000 | 0.530757000 | -1.291486000 |
| H | -2.612273000 | 1.514404000 | 1.961836000 |
| H | -2.611379000 | 0.941989000 | -2.292322000 |
| C | -2.600488000 | 1.383893000 | -0.186161000 |
| H | -2.614901000 | 2.456268000 | -0.330456000 |
| H | -2.614978000 | -2.456254000 | 0.330467000 |
| C | 2.598908000 | 0.853286000 | 1.105253000 |
| H | 2.612342000 | 1.514403000 | 1.961826000 |
| C | 2.600512000 | 1.383879000 | -0.186176000 |
| H | 2.614966000 | 2.456253000 | -0.330477000 |
| H | 2.611390000 | 0.941987000 | -2.292322000 |
| C | 2.598333000 | 0.530741000 | -1.291494000 |
| C | 2.598904000 | -0.853300000 | -1.105249000 |
| C | 2.598323000 | -0.530750000 | 1.291490000 |
| C | 2.600490000 | -1.383892000 | 0.186168000 |
| H | 2.611352000 | -0.941978000 | 2.292328000 |
| H | 2.614907000 | -2.456267000 | 0.330469000 |
| H | 2.612295000 | -1.514412000 | -1.961828000 |

Electronic Transitions

| Wavelength, nm | Oscillator strength |
|---|---|
| 6143.25 | 0.0000 |
| 4993.48 | 0.0000 |
| 2744.06 | 0.0000 |
| 2735.57 | 0.0000 |
| 425.65 | 0.0000 |
| 424.58 | 0.0000 |
| 422.98 | 0.0000 |
| 422.88 | 0.0000 |
| 408.40 | 0.0004 |
| 408.35 | 0.0004 |
| 405.39 | 0.0000 |
| 405.37 | 0.0000 |
| 376.47 | 0.0000 |
| 375.06 | 0.0000 |
| 370.70 | 0.0000 |
| 368.95 | 0.0000 |
| 364.30 | 0.0000 |
| 364.10 | 0.0000 |
| 361.88 | 0.0000 |
| 350.07 | 0.1308 |
| 322.62 | 0.0000 |
| 320.59 | 0.0001 |
| 318.88 | 0.0000 |
| 312.14 | 0.0000 |
| 308.98 | 0.0000 |
| 294.74 | 0.0046 |
| 294.52 | 0.0000 |
| 290.68 | 0.0128 |
| 288.88 | 0.0080 |
| 283.18 | 0.0000 |
| 283.13 | 0.0000 |
| 268.26 | 0.0207 |
| 267.96 | 0.0214 |
| 250.98 | 0.0000 |
| 250.57 | 0.0000 |
| 249.76 | 0.0003 |
| 249.30 | 0.0005 |
| 238.22 | 0.0000 |
| 237.51 | 0.0000 |
| 235.96 | 0.0003 |
| 235.21 | 0.0000 |
| 235.17 | 0.0000 |
| 235.13 | 0.0000 |
| 235.08 | 0.0000 |

| | |
|---|---|
| 220.76 | 0.0000 |
| 220.42 | 0.0001 |
| 220.39 | 0.0000 |
| 220.32 | 0.0000 |
| 220.28 | 0.0000 |
| 220.27 | 0.0000 |
| 220.21 | 0.0000 |
| 220.20 | 0.0000 |
| 219.70 | 0.0000 |
| 219.22 | 0.0004 |
| 216.69 | 0.1439 |
| 212.71 | 0.0000 |
| 211.60 | 0.0000 |
| 209.89 | 0.0003 |
| 209.37 | 0.0000 |
| 204.33 | 0.0000 |
| 203.19 | 0.0567 |
| 201.33 | 0.0003 |
| 201.31 | 0.3462 |
| 200.54 | 0.0000 |
| 200.53 | 0.0000 |
| 200.16 | 0.0019 |
| 200.12 | 0.0021 |
| 199.67 | 0.0002 |
| 198.44 | 0.0024 |
| 198.40 | 0.0000 |
| 198.37 | 0.0000 |
| 198.31 | 0.0000 |
| 198.27 | 0.0000 |
| 197.33 | 0.0007 |
| 196.31 | 0.0000 |
| 196.10 | 0.0000 |
| 195.97 | 0.0000 |
| 195.85 | 0.0011 |
| 195.44 | 0.0005 |
| 194.32 | 0.0000 |
| 193.46 | 0.0000 |
| 193.42 | 0.0000 |
| 193.39 | 0.0000 |
| 193.31 | 0.0000 |
| 191.42 | 0.0055 |
| 191.39 | 0.0056 |
| 187.55 | 0.0000 |
| 187.52 | 0.0000 |
| 187.50 | 0.0000 |
| 187.46 | 0.0000 |

| | |
|---|---|
| 186.44 | 0.0000 |
| 186.41 | 0.0000 |
| 182.15 | 0.0102 |
| 182.14 | 0.0103 |
| 182.05 | 0.0000 |
| 182.02 | 0.0000 |
| 177.36 | 0.0000 |
| 176.92 | 0.0000 |
| 174.89 | 0.2345 |
| 174.73 | 0.0000 |

$Fe^{+}(benzene)_2$
M06-L
m = 2

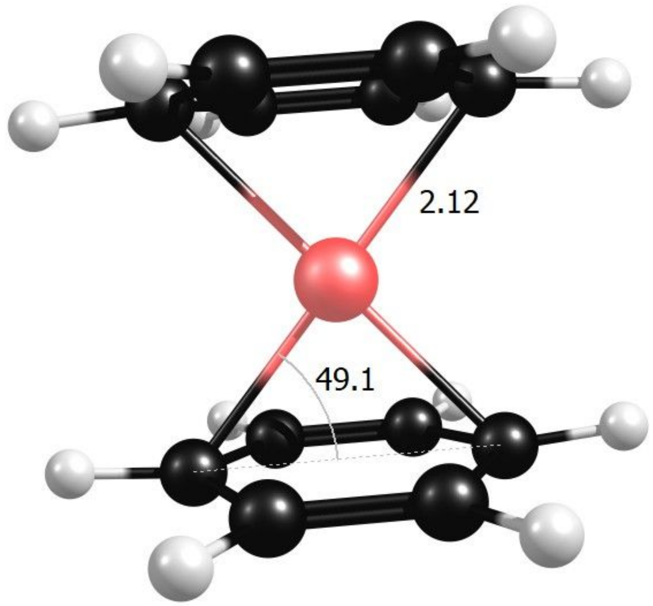


Benzene Dihedral: 6°
Coordinates:

| | | | |
|---|---|---|---|
| Fe | 0.000000000 | -0.000004000 | -0.000012000 |
| C | 1.665115000 | -1.229858000 | 0.697341000 |
| C | 1.665143000 | -1.229969000 | -0.697149000 |
| C | 1.598541000 | 0.000108000 | 1.386653000 |
| H | 1.645596000 | -2.159882000 | -1.248549000 |
| H | 1.515695000 | 0.000185000 | 2.465326000 |
| C | 1.598575000 | -0.000108000 | -1.386644000 |
| C | 1.665130000 | 1.229968000 | 0.697159000 |
| H | 1.515763000 | -0.000185000 | -2.465319000 |
| H | 1.645572000 | 2.159881000 | 1.248560000 |
| C | 1.665138000 | 1.229858000 | -0.697330000 |
| H | 1.645581000 | 2.159683000 | -1.248879000 |
| H | 1.645534000 | -2.159683000 | 1.248889000 |
| C | -1.598570000 | -0.000108000 | -1.386646000 |
| H | -1.515758000 | -0.000192000 | -2.465322000 |
| C | -1.665130000 | 1.229861000 | -0.697337000 |
| H | -1.645561000 | 2.159685000 | -1.248887000 |
| H | -1.645564000 | 2.159883000 | 1.248555000 |
| C | -1.665131000 | 1.229972000 | 0.697152000 |
| C | -1.598545000 | 0.000113000 | 1.386650000 |
| C | -1.665147000 | -1.229967000 | -0.697147000 |
| C | -1.665121000 | -1.229855000 | 0.697342000 |
| H | -1.645594000 | -2.159879000 | -1.248548000 |
| H | -1.645539000 | -2.159678000 | 1.248893000 |
| H | -1.515705000 | 0.000197000 | 2.465323000 |

Electronic Transitions

| Wavelength, nm | Oscillator strength |
|---|---|
| 1778.46 | 0.0000 |
| 728.68 | 0.0000 |
| 622.07 | 0.0000 |
| 551.74 | 0.0000 |
| 512.86 | 0.0000 |
| 498.36 | 0.0077 |
| 492.65 | 0.0000 |
| 485.82 | 0.0064 |
| 465.42 | 0.0000 |
| 439.48 | 0.0000 |
| 426.13 | 0.0000 |
| 420.12 | 0.0000 |
| 369.34 | 0.0000 |
| 358.74 | 0.0020 |
| 347.82 | 0.0000 |
| 338.44 | 0.0000 |
| 332.46 | 0.0000 |
| 331.82 | 0.0000 |
| 330.31 | 0.0000 |
| 325.89 | 0.0058 |
| 315.61 | 0.0000 |
| 313.64 | 0.0176 |
| 308.24 | 0.0000 |
| 307.37 | 0.0000 |
| 298.80 | 0.0000 |
| 290.99 | 0.0000 |
| 288.52 | 0.0213 |
| 285.53 | 0.0000 |
| 282.96 | 0.0000 |
| 282.62 | 0.0000 |
| 269.91 | 0.0000 |
| 269.85 | 0.0000 |
| 266.50 | 0.0000 |
| 260.71 | 0.0000 |
| 259.63 | 0.0074 |
| 256.59 | 0.0000 |
| 250.03 | 0.0049 |
| 248.90 | 0.0000 |
| 246.26 | 0.0000 |
| 244.71 | 0.0000 |
| 243.30 | 0.0000 |
| 233.26 | 0.1432 |
| 225.24 | 0.0000 |
| 223.93 | 0.0000 |

| | |
|---|---|
| 223.84 | 0.0004 |
| 223.62 | 0.0000 |
| 222.97 | 0.0053 |
| 222.95 | 0.0000 |
| 217.57 | 0.0000 |
| 212.79 | 0.0000 |
| 210.89 | 0.0000 |
| 210.80 | 0.0000 |
| 209.85 | 0.0014 |
| 207.33 | 0.0025 |
| 206.03 | 0.0000 |
| 205.86 | 0.0000 |
| 204.48 | 0.0012 |
| 203.08 | 0.0000 |
| 202.82 | 0.0011 |
| 202.81 | 0.0000 |
| 200.02 | 0.0000 |
| 199.90 | 0.1668 |
| 199.07 | 0.0000 |
| 197.98 | 0.0000 |
| 195.73 | 0.0000 |
| 194.48 | 0.0015 |
| 191.70 | 0.0000 |
| 191.68 | 0.0000 |
| 188.45 | 0.0000 |
| 187.89 | 0.0001 |
| 187.85 | 0.0019 |
| 187.82 | 0.0004 |
| 187.02 | 0.0000 |
| 186.95 | 0.0000 |
| 185.88 | 0.0000 |
| 185.43 | 0.0000 |
| 185.30 | 0.0002 |
| 185.02 | 0.0032 |
| 184.67 | 0.0002 |
| 184.66 | 0.0000 |
| 184.62 | 0.0000 |
| 184.06 | 0.0000 |
| 183.78 | 0.0016 |
| 183.69 | 0.0000 |
| 183.59 | 0.0000 |
| 183.14 | 0.0000 |
| 183.01 | 0.0001 |
| 182.94 | 0.0007 |
| 182.59 | 0.0008 |
| 182.12 | 0.3871 |

| | |
|---|---|
| 181.81 | 0.0000 |
| 181.26 | 0.0005 |
| 180.81 | 0.0056 |
| 180.66 | 0.0000 |
| 180.65 | 0.0000 |
| 179.89 | 0.0000 |
| 179.76 | 0.0000 |
| 178.86 | 0.0077 |
| 178.63 | 0.0036 |
| 178.42 | 0.0000 |

Fe$^{+}$(benzene)$_2$
M06-L
m = 4

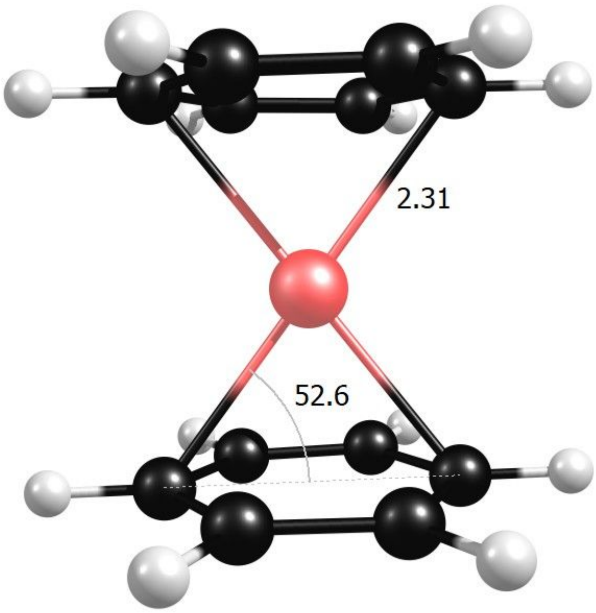


Benzene Dihedral: 3°
Coordinates:

| | | | |
|---|---|---|---|
| Fe | 0.000000000 | 0.000000000 | 0.000000000 |
| C | 0.723001000 | -2.199023000 | -0.010011000 |
| C | 0.044395000 | -1.998373000 | 1.200174000 |
| C | 0.058047000 | -1.962479000 | -1.225337000 |
| H | 0.561683000 | -2.164988000 | 2.136063000 |
| H | 0.564488000 | -2.136684000 | -2.164087000 |
| C | -1.269664000 | -1.510006000 | 1.213966000 |
| C | -1.275424000 | -1.529648000 | -1.211730000 |
| H | -1.776997000 | -1.338410000 | 2.152721000 |
| H | -1.782179000 | -1.332603000 | -2.147469000 |
| C | -1.934845000 | -1.274159000 | -0.001268000 |
| H | -2.956108000 | -0.920461000 | -0.001504000 |
| H | 1.743059000 | -2.556162000 | -0.009784000 |
| C | -0.058047000 | 1.962479000 | 1.225337000 |
| H | -0.564488000 | 2.136684000 | 2.164087000 |
| C | -0.723001000 | 2.199023000 | 0.010011000 |
| H | -1.743059000 | 2.556162000 | 0.009784000 |
| H | -0.561683000 | 2.164988000 | -2.136063000 |
| C | -0.044395000 | 1.998373000 | -1.200174000 |
| C | 1.269664000 | 1.510006000 | -1.213966000 |
| C | 1.275424000 | 1.529648000 | 1.211730000 |
| C | 1.934845000 | 1.274159000 | 0.001268000 |
| H | 1.782179000 | 1.332603000 | 2.147469000 |
| H | 2.956108000 | 0.920461000 | 0.001504000 |
| H | 1.776997000 | 1.338410000 | -2.152721000 |

Electronic Transitions

| Wavelength, nm | Oscillator strength |
|---|---|
| 2961.00 | 0.0000 |
| 1618.64 | 0.0000 |
| 679.95 | 0.0000 |
| 611.23 | 0.0000 |
| 514.62 | 0.0000 |
| 480.79 | 0.0000 |
| 463.07 | 0.0090 |
| 414.70 | 0.0000 |
| 410.56 | 0.0000 |
| 393.49 | 0.0000 |
| 387.05 | 0.0000 |
| 384.92 | 0.1288 |
| 381.52 | 0.0000 |
| 377.82 | 0.0000 |
| 375.74 | 0.0037 |
| 360.16 | 0.0073 |
| 347.11 | 0.0000 |
| 342.37 | 0.0000 |
| 340.85 | 0.0000 |
| 335.97 | 0.0096 |
| 334.65 | 0.0000 |
| 324.53 | 0.0099 |
| 291.19 | 0.0146 |
| 290.25 | 0.0000 |
| 288.45 | 0.0000 |
| 274.28 | 0.0051 |
| 261.06 | 0.0000 |
| 258.20 | 0.0000 |
| 251.60 | 0.0000 |
| 250.80 | 0.0000 |
| 250.27 | 0.0149 |
| 243.49 | 0.0000 |
| 233.96 | 0.0000 |
| 233.52 | 0.0000 |
| 232.39 | 0.0004 |
| 232.07 | 0.0000 |
| 231.63 | 0.0265 |
| 231.19 | 0.0000 |
| 226.64 | 0.0000 |
| 223.19 | 0.0000 |
| 221.08 | 0.0000 |
| 219.71 | 0.0000 |
| 218.31 | 0.0000 |
| 217.20 | 0.0146 |

| | |
|---|---|
| 216.75 | 0.0000 |
| 215.79 | 0.0000 |
| 215.48 | 0.0003 |
| 214.79 | 0.0000 |
| 214.29 | 0.0003 |
| 214.14 | 0.1255 |
| 212.06 | 0.0000 |
| 211.37 | 0.0000 |
| 208.67 | 0.0000 |
| 206.55 | 0.0000 |
| 204.76 | 0.0000 |
| 204.76 | 0.0000 |
| 203.33 | 0.0000 |
| 203.11 | 0.0004 |
| 203.02 | 0.0000 |
| 201.79 | 0.0014 |
| 199.23 | 0.0000 |
| 198.41 | 0.0000 |
| 198.34 | 0.0333 |
| 197.24 | 0.0000 |
| 196.74 | 0.0000 |
| 196.37 | 0.0030 |
| 194.76 | 0.0038 |
| 194.67 | 0.0043 |
| 194.52 | 0.0000 |
| 193.95 | 0.0002 |
| 191.52 | 0.0000 |
| 190.45 | 0.0004 |
| 187.94 | 0.0000 |
| 187.69 | 0.0376 |
| 187.57 | 0.0000 |
| 187.34 | 0.0000 |
| 187.31 | 0.0250 |
| 186.63 | 0.0047 |
| 185.41 | 0.0004 |
| 185.06 | 0.0000 |
| 185.00 | 0.0000 |
| 184.56 | 0.0000 |
| 184.28 | 0.0052 |
| 183.64 | 0.0000 |
| 183.53 | 0.0009 |
| 182.61 | 0.0139 |
| 182.30 | 0.0000 |
| 182.13 | 0.0000 |
| 181.46 | 0.0013 |
| 180.90 | 0.1268 |

| | |
|---|---|
| 180.55 | 0.0000 |
| 180.07 | 0.0030 |
| 179.65 | 0.0000 |
| 179.51 | 0.0003 |
| 179.28 | 0.0000 |
| 178.45 | 0.0000 |
| 177.33 | 0.0000 |
| 177.22 | 0.0030 |
| 176.89 | 0.0129 |
| 176.58 | 0.0000 |

$Fe^{+}(benzene)_2$
M06-L
m = 6

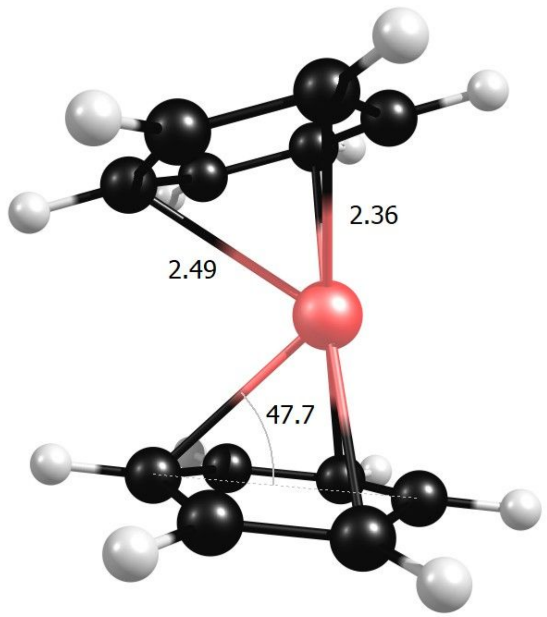


Benzene Dihedral: 12°
Coordinates:

| | | | |
|---|---|---|---|
| Fe | -0.000008000 | 0.454522000 | 0.000016000 |
| C | -1.759190000 | -0.826507000 | -1.224568000 |
| C | -1.529162000 | -1.510289000 | -0.000283000 |
| C | -2.011785000 | 0.530575000 | -1.229836000 |
| H | -1.324060000 | -2.569632000 | -0.000501000 |
| H | -2.150135000 | 1.057676000 | -2.164142000 |
| C | -1.759072000 | -0.826980000 | 1.224294000 |
| C | -2.009476000 | 1.263019000 | 0.000279000 |
| H | -1.703261000 | -1.368707000 | 2.159080000 |
| H | -2.216184000 | 2.323083000 | 0.000501000 |
| C | -2.011658000 | 0.530097000 | 1.230108000 |
| H | -2.149897000 | 1.056844000 | 2.164630000 |
| H | -1.703466000 | -1.367869000 | -2.159571000 |
| C | 1.759103000 | -0.826966000 | 1.224285000 |
| H | 1.703301000 | -1.368690000 | 2.159073000 |
| C | 2.011659000 | 0.530119000 | 1.230097000 |
| H | 2.149890000 | 1.056868000 | 2.164619000 |
| H | 2.216148000 | 2.323105000 | 0.000485000 |
| C | 2.009461000 | 1.263037000 | 0.000268000 |
| C | 2.011756000 | 0.530594000 | -1.229845000 |
| C | 1.529205000 | -1.510281000 | -0.000286000 |
| C | 1.759194000 | -0.826495000 | -1.224575000 |
| H | 1.324128000 | -2.569629000 | -0.000498000 |
| H | 1.703467000 | -1.367855000 | -2.159578000 |
| H | 2.150049000 | 1.057704000 | -2.164155000 |

Electronic Transitions

| Wavelength, nm | Oscillator strength |
|---|---|
| 2017.01 | 0.0001 |
| 1612.96 | 0.0001 |
| 1192.65 | 0.0459 |
| 1093.80 | 0.0001 |
| 1011.22 | 0.0000 |
| 704.80 | 0.0018 |
| 647.03 | 0.0000 |
| 458.54 | 0.0052 |
| 385.21 | 0.0163 |
| 372.00 | 0.0000 |
| 341.83 | 0.0001 |
| 340.93 | 0.0000 |
| 340.61 | 0.0034 |
| 338.82 | 0.0002 |
| 334.62 | 0.0001 |
| 333.26 | 0.0003 |
| 327.81 | 0.0001 |
| 323.58 | 0.0004 |
| 316.83 | 0.0010 |
| 308.48 | 0.0005 |
| 304.88 | 0.0002 |
| 300.47 | 0.0002 |
| 297.18 | 0.0000 |
| 295.92 | 0.0030 |
| 295.84 | 0.0000 |
| 291.85 | 0.0000 |
| 290.23 | 0.0018 |
| 289.88 | 0.0221 |
| 287.39 | 0.0034 |
| 283.18 | 0.0841 |
| 280.83 | 0.0117 |
| 279.59 | 0.0000 |
| 270.01 | 0.0000 |
| 264.56 | 0.1179 |
| 263.30 | 0.0004 |
| 262.39 | 0.0039 |
| 258.21 | 0.0055 |
| 257.56 | 0.0076 |
| 255.11 | 0.0000 |
| 252.39 | 0.0034 |
| 249.73 | 0.0852 |
| 246.78 | 0.0004 |
| 241.95 | 0.0065 |
| 239.62 | 0.0019 |

| | |
|---|---|
| 239.05 | 0.0086 |
| 236.07 | 0.0033 |
| 234.88 | 0.0045 |
| 233.24 | 0.0004 |
| 232.94 | 0.0107 |
| 232.51 | 0.0006 |
| 232.36 | 0.0000 |
| 231.91 | 0.0000 |
| 227.29 | 0.0000 |
| 226.40 | 0.0000 |
| 225.33 | 0.0000 |
| 221.98 | 0.0000 |
| 221.61 | 0.0005 |
| 220.20 | 0.0009 |
| 218.39 | 0.0002 |
| 217.98 | 0.0007 |
| 217.62 | 0.0003 |
| 217.45 | 0.0023 |
| 216.05 | 0.0001 |
| 215.61 | 0.0010 |
| 213.06 | 0.0047 |
| 212.74 | 0.0000 |
| 210.92 | 0.0000 |
| 210.38 | 0.0000 |
| 209.14 | 0.0000 |
| 207.86 | 0.0000 |
| 207.76 | 0.0003 |
| 205.96 | 0.0178 |
| 203.49 | 0.0000 |
| 202.82 | 0.0000 |
| 202.54 | 0.0000 |
| 201.28 | 0.0188 |
| 199.98 | 0.0015 |
| 199.85 | 0.0025 |
| 199.65 | 0.0000 |
| 196.19 | 0.0030 |
| 196.16 | 0.0000 |
| 195.66 | 0.0051 |
| 195.44 | 0.0000 |
| 194.91 | 0.0000 |
| 194.60 | 0.0455 |
| 192.96 | 0.0000 |
| 190.76 | 0.0038 |
| 190.71 | 0.0005 |
| 189.57 | 0.0018 |
| 189.47 | 0.0000 |

| | |
|---|---|
| 186.96 | 0.0042 |
| 186.81 | 0.0033 |
| 186.72 | 0.0051 |
| 185.95 | 0.0121 |
| 185.95 | 0.0002 |
| 183.32 | 0.0111 |
| 183.30 | 0.0000 |
| 181.92 | 0.0005 |
| 179.44 | 0.0127 |
| 179.24 | 0.1317 |

$Fe^{+}$(benzene)$_2$
MN15-L
m = 2

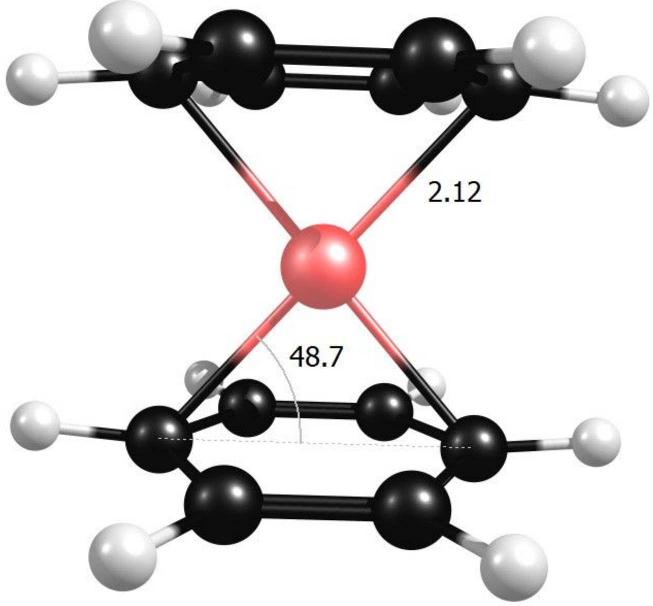


Benzene Dihedral: 6°
Coordinates:

| | | | |
|---|---|---|---|
| Fe | 0.000000000 | 0.000000000 | 0.000000000 |
| C | 0.792781000 | -2.037612000 | 0.008708000 |
| C | 0.130570000 | -1.804296000 | 1.227949000 |
| C | 0.127685000 | -1.731629000 | -1.213151000 |
| H | 0.644507000 | -1.957228000 | 2.180257000 |
| H | 0.671277000 | -1.825623000 | -2.157494000 |
| C | -1.188877000 | -1.267862000 | 1.210829000 |
| C | -1.235641000 | -1.319230000 | -1.230322000 |
| H | -1.671164000 | -1.000519000 | 2.155301000 |
| H | -1.730970000 | -1.113780000 | -2.182611000 |
| C | -1.897865000 | -1.086014000 | -0.011116000 |
| H | -2.918508000 | -0.695542000 | 0.003728000 |
| H | 1.831965000 | -2.375691000 | -0.006068000 |
| C | -0.127685000 | 1.731629000 | 1.213151000 |
| H | -0.671277000 | 1.825623000 | 2.157494000 |
| C | -0.792781000 | 2.037612000 | -0.008708000 |
| H | -1.831965000 | 2.375691000 | 0.006068000 |
| H | -0.644507000 | 1.957228000 | -2.180257000 |
| C | -0.130570000 | 1.804296000 | -1.227949000 |
| C | 1.188877000 | 1.267862000 | -1.210829000 |
| C | 1.235641000 | 1.319230000 | 1.230322000 |
| C | 1.897865000 | 1.086014000 | 0.011116000 |
| H | 1.730970000 | 1.113780000 | 2.182611000 |
| H | 2.918508000 | 0.695542000 | -0.003728000 |
| H | 1.671164000 | 1.000519000 | -2.155301000 |

Electronic Transitions

| Wavelength, nm | Oscillator strength |
|---|---|
| 2228.82 | 0.0000 |
| 542.24 | 0.0000 |
| 497.48 | 0.0000 |
| 488.93 | 0.0000 |
| 478.57 | 0.0000 |
| 456.79 | 0.0101 |
| 451.07 | 0.0085 |
| 439.75 | 0.0000 |
| 437.33 | 0.0000 |
| 418.29 | 0.0000 |
| 407.64 | 0.0000 |
| 401.32 | 0.0000 |
| 366.45 | 0.0000 |
| 359.92 | 0.0044 |
| 338.22 | 0.0000 |
| 331.65 | 0.0000 |
| 325.31 | 0.0000 |
| 324.12 | 0.0000 |
| 322.53 | 0.0000 |
| 308.99 | 0.0000 |
| 308.95 | 0.0091 |
| 302.05 | 0.0000 |
| 294.82 | 0.0357 |
| 292.78 | 0.0000 |
| 290.40 | 0.0000 |
| 283.76 | 0.0000 |
| 270.97 | 0.0000 |
| 257.11 | 0.0000 |
| 253.98 | 0.0000 |
| 251.99 | 0.0000 |
| 249.61 | 0.0000 |
| 247.72 | 0.0340 |
| 246.87 | 0.0000 |
| 244.78 | 0.0000 |
| 241.34 | 0.0199 |
| 240.20 | 0.0000 |
| 239.30 | 0.0000 |
| 238.58 | 0.0000 |
| 237.23 | 0.0000 |
| 233.15 | 0.0000 |
| 230.14 | 0.0151 |
| 227.50 | 0.0093 |
| 225.74 | 0.0000 |
| 222.50 | 0.0000 |

| | |
|---|---|
| 221.41 | 0.0000 |
| 219.81 | 0.0117 |
| 216.89 | 0.1578 |
| 215.67 | 0.0000 |
| 212.79 | 0.0000 |
| 212.27 | 0.0000 |
| 210.43 | 0.0000 |
| 209.81 | 0.0001 |
| 207.35 | 0.0000 |
| 206.93 | 0.0019 |
| 204.82 | 0.0011 |
| 203.01 | 0.0000 |
| 200.66 | 0.0000 |
| 198.48 | 0.0000 |
| 198.32 | 0.0000 |
| 197.93 | 0.0033 |
| 197.77 | 0.0000 |
| 196.91 | 0.0115 |
| 194.81 | 0.0000 |
| 193.64 | 0.0000 |
| 191.91 | 0.0156 |
| 191.59 | 0.0000 |
| 190.85 | 0.0000 |
| 189.79 | 0.0000 |
| 188.94 | 0.0119 |
| 187.24 | 0.0000 |
| 186.82 | 0.0650 |
| 186.65 | 0.0000 |
| 186.58 | 0.0018 |
| 185.95 | 0.0000 |
| 185.64 | 0.0000 |
| 184.89 | 0.0008 |
| 183.09 | 0.0005 |
| 182.95 | 0.0000 |
| 182.39 | 0.0000 |
| 182.37 | 0.0000 |
| 180.48 | 0.0000 |
| 180.36 | 0.0002 |
| 180.17 | 0.0000 |
| 179.68 | 0.0000 |
| 178.83 | 0.0000 |
| 178.83 | 0.0002 |
| 178.32 | 0.0000 |
| 178.07 | 0.0161 |
| 177.47 | 0.0000 |
| 177.44 | 0.2508 |

| | |
|---|---|
| 176.88 | 0.0000 |
| 176.42 | 0.0000 |
| 176.15 | 0.0000 |
| 176.07 | 0.0138 |
| 175.89 | 0.0000 |
| 175.85 | 0.0000 |
| 175.62 | 0.0000 |
| 175.29 | 0.0000 |
| 175.27 | 0.2365 |
| 175.14 | 0.0000 |

$Fe^{+}(benzene)_2$
MN15-L
m = 4

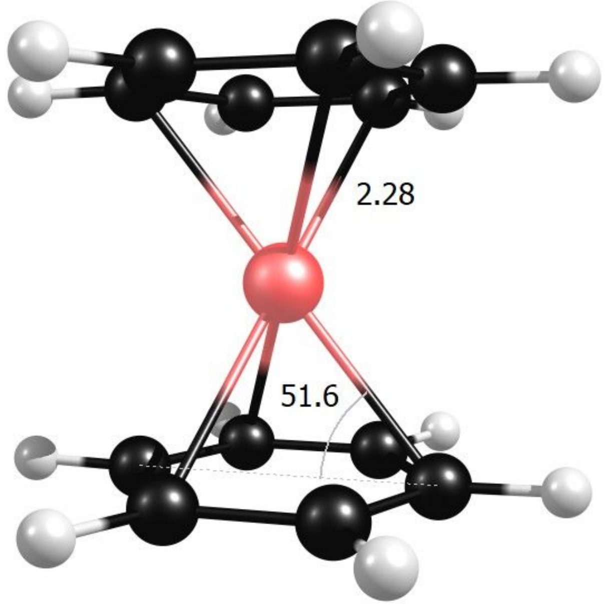


Benzene Dihedral: 0°
Coordinates:

| | | | |
|---|---|---|---|
| Fe | 0.000000000 | 0.000000000 | 0.000000000 |
| C | 0.740519000 | -2.161287000 | -0.002754000 |
| C | 0.072731000 | -1.925873000 | 1.226937000 |
| C | 0.070513000 | -1.923429000 | -1.230768000 |
| H | 0.595352000 | -2.089143000 | 2.172128000 |
| H | 0.591432000 | -2.084831000 | -2.177219000 |
| C | -1.265100000 | -1.452808000 | 1.228634000 |
| C | -1.267320000 | -1.450358000 | -1.229115000 |
| H | -1.772054000 | -1.251970000 | 2.175120000 |
| H | -1.775969000 | -1.247633000 | -2.174288000 |
| C | -1.935151000 | -1.215022000 | 0.000599000 |
| H | -2.957834000 | -0.831248000 | 0.001904000 |
| H | 1.777109000 | -2.505745000 | -0.004030000 |
| C | -0.070513000 | 1.923429000 | 1.230768000 |
| H | -0.591432000 | 2.084831000 | 2.177219000 |
| C | -0.740519000 | 2.161287000 | 0.002754000 |
| H | -1.777109000 | 2.505745000 | 0.004030000 |
| H | -0.595352000 | 2.089143000 | -2.172128000 |
| C | -0.072731000 | 1.925873000 | -1.226937000 |
| C | 1.265100000 | 1.452808000 | -1.228634000 |
| C | 1.267320000 | 1.450358000 | 1.229115000 |
| C | 1.935151000 | 1.215022000 | -0.000599000 |
| H | 1.775969000 | 1.247633000 | 2.174288000 |
| H | 2.957834000 | 0.831248000 | -0.001904000 |
| H | 1.772054000 | 1.251970000 | -2.175120000 |

Electronic Transitions

| Wavelength, nm | Oscillator strength |
|---|---|
| 1714.10 | 0.0000 |
| 1713.75 | 0.0000 |
| 567.40 | 0.0000 |
| 557.82 | 0.0000 |
| 445.38 | 0.0000 |
| 445.37 | 0.0000 |
| 402.24 | 0.0000 |
| 402.24 | 0.0000 |
| 392.47 | 0.0000 |
| 380.40 | 0.0000 |
| 380.40 | 0.0000 |
| 341.46 | 0.2336 |
| 340.26 | 0.0000 |
| 324.13 | 0.0000 |
| 322.04 | 0.0000 |
| 315.83 | 0.0000 |
| 315.06 | 0.0000 |
| 315.06 | 0.0000 |
| 303.16 | 0.0000 |
| 294.65 | 0.0000 |
| 291.38 | 0.0299 |
| 291.38 | 0.0299 |
| 286.16 | 0.0000 |
| 286.16 | 0.0000 |
| 261.85 | 0.0000 |
| 261.84 | 0.0000 |
| 256.51 | 0.0000 |
| 256.50 | 0.0000 |
| 214.00 | 0.0000 |
| 212.70 | 0.0000 |
| 212.69 | 0.0000 |
| 210.16 | 0.0000 |
| 209.25 | 0.0000 |
| 209.25 | 0.0000 |
| 208.34 | 0.0000 |
| 208.34 | 0.0000 |
| 207.72 | 0.0000 |
| 207.53 | 0.0000 |
| 207.52 | 0.0000 |
| 206.83 | 0.0000 |
| 206.83 | 0.0000 |
| 206.20 | 0.0000 |
| 206.19 | 0.0000 |
| 204.01 | 0.0001 |

| | |
|---|---|
| 204.01 | 0.0001 |
| 203.58 | 0.0000 |
| 197.80 | 0.0000 |
| 197.56 | 0.0000 |
| 195.97 | 0.0000 |
| 195.09 | 0.0006 |
| 195.09 | 0.0006 |
| 194.99 | 0.0000 |
| 194.98 | 0.0000 |
| 194.98 | 0.0000 |
| 193.68 | 0.0000 |
| 193.68 | 0.0000 |
| 192.90 | 0.0000 |
| 192.32 | 0.0000 |
| 192.18 | 0.0000 |
| 190.79 | 0.0000 |
| 190.79 | 0.0000 |
| 190.70 | 0.0000 |
| 190.70 | 0.0000 |
| 190.01 | 0.0000 |
| 190.01 | 0.0000 |
| 188.23 | 0.0000 |
| 186.97 | 0.0000 |
| 186.97 | 0.0000 |
| 184.56 | 0.1122 |
| 182.93 | 0.0000 |
| 181.09 | 0.2434 |
| 178.97 | 0.0001 |
| 178.96 | 0.0001 |
| 178.46 | 0.0000 |
| 176.71 | 0.0000 |
| 176.71 | 0.0000 |
| 176.70 | 0.0920 |
| 176.69 | 0.0920 |
| 176.09 | 0.0000 |
| 175.52 | 0.0000 |
| 175.52 | 0.0000 |
| 175.41 | 0.0000 |
| 174.90 | 0.0000 |
| 173.65 | 0.0000 |
| 172.81 | 0.0000 |
| 172.81 | 0.0000 |
| 169.49 | 0.0000 |
| 169.49 | 0.0000 |
| 168.96 | 0.0000 |
| 168.82 | 0.062 |

| | |
|---|---|
| 168.76 | 0.0003 |
| 168.76 | 0.0000 |
| 168.61 | 0.0000 |
| 168.61 | 0.0000 |
| 168.52 | 0.0000 |
| 168.36 | 0.0000 |
| 167.97 | 0.0000 |
| 167.85 | 0.0000 |
| 167.85 | 0.0000 |
| 167.77 | 0.0000 |

$Fe^{+}(benzene)_2$
MN15-L
m = 6

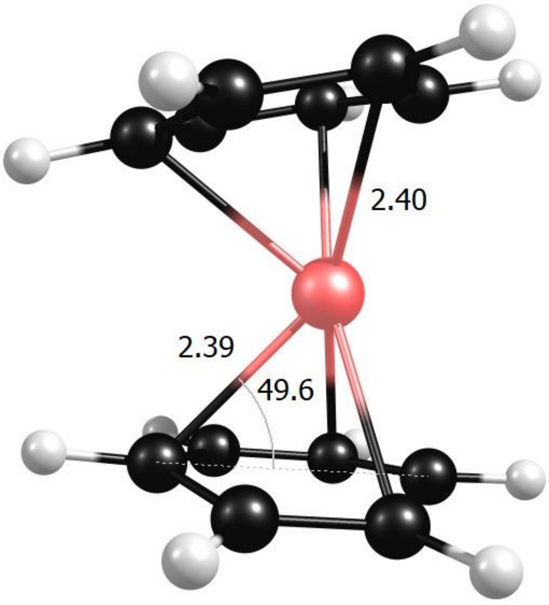


Benzene Dihedral: 12°
Coordinates:

| | | | |
|---|---|---|---|
| Fe | 0.000000000 | 0.000005000 | -0.305537000 |
| C | -1.591243000 | -0.000012000 | 1.479946000 |
| C | -1.827618000 | 1.237085000 | 0.791375000 |
| C | -1.827614000 | -1.237099000 | 0.791356000 |
| H | -1.800913000 | 2.179872000 | 1.344229000 |
| H | -1.800905000 | -2.179894000 | 1.344196000 |
| C | -2.031611000 | 1.239792000 | -0.587896000 |
| C | -2.031606000 | -1.239786000 | -0.587915000 |
| H | -2.157976000 | 2.182109000 | -1.127444000 |
| H | -2.157967000 | -2.182095000 | -1.127477000 |
| C | -1.978304000 | 0.000009000 | -1.321734000 |
| H | -2.108960000 | 0.000017000 | -2.404871000 |
| H | -1.395149000 | -0.000019000 | 2.552509000 |
| C | 2.031611000 | 1.239790000 | -0.587898000 |
| H | 2.157976000 | 2.182106000 | -1.127449000 |
| C | 1.978306000 | 0.000005000 | -1.321732000 |
| H | 2.108962000 | 0.000010000 | -2.404869000 |
| H | 2.157965000 | -2.182098000 | -1.127470000 |
| C | 2.031606000 | -1.239787000 | -0.587909000 |
| C | 1.827613000 | -1.237096000 | 0.791362000 |
| C | 1.827618000 | 1.237087000 | 0.791373000 |
| C | 1.591242000 | -0.000007000 | 1.479947000 |
| H | 1.800911000 | 2.179876000 | 1.344224000 |
| H | 1.395147000 | -0.000012000 | 2.552511000 |
| H | 1.800902000 | -2.179890000 | 1.344204000 |

Electronic Transitions

| Wavelength, nm | Oscillator strength |
| --- | --- |
| 1907.39 | 0.0000 |
| 1543.08 | 0.0000 |
| 1106.80 | 0.0605 |
| 1016.17 | 0.0000 |
| 982.72 | 0.0000 |
| 571.78 | 0.0000 |
| 546.08 | 0.0009 |
| 528.45 | 0.0043 |
| 364.00 | 0.0003 |
| 341.62 | 0.0085 |
| 337.13 | 0.0000 |
| 319.68 | 0.0015 |
| 316.25 | 0.0000 |
| 312.56 | 0.0000 |
| 309.03 | 0.0001 |
| 307.82 | 0.0000 |
| 307.41 | 0.0013 |
| 304.92 | 0.0007 |
| 295.76 | 0.0634 |
| 294.72 | 0.0002 |
| 293.33 | 0.0026 |
| 285.53 | 0.0003 |
| 284.96 | 0.0229 |
| 281.53 | 0.0038 |
| 280.40 | 0.0000 |
| 277.85 | 0.0140 |
| 277.28 | 0.0002 |
| 276.32 | 0.0000 |
| 268.31 | 0.0018 |
| 264.91 | 0.0029 |
| 262.19 | 0.0079 |
| 256.72 | 0.0015 |
| 251.26 | 0.0000 |
| 250.30 | 0.0002 |
| 249.71 | 0.0360 |
| 248.54 | 0.0220 |
| 245.06 | 0.0000 |
| 243.39 | 0.0030 |
| 242.88 | 0.0824 |
| 240.61 | 0.0119 |
| 235.22 | 0.0000 |
| 233.83 | 0.1435 |
| 230.91 | 0.0000 |
| 229.03 | 0.0048 |

| | |
|---|---|
| 228.98 | 0.0006 |
| 227.2 | 0.0044 |
| 226.10 | 0.0035 |
| 224.63 | 0.0006 |
| 223.99 | 0.0001 |
| 222.18 | 0.0074 |
| 221.33 | 0.0000 |
| 220.37 | 0.0033 |
| 220.30 | 0.0003 |
| 216.71 | 0.0000 |
| 212.66 | 0.0000 |
| 212.43 | 0.0060 |
| 210.25 | 0.0000 |
| 208.40 | 0.0031 |
| 207.08 | 0.0000 |
| 205.58 | 0.0012 |
| 204.81 | 0.0128 |
| 204.72 | 0.0000 |
| 203.39 | 0.0010 |
| 199.45 | 0.0000 |
| 199.13 | 0.0004 |
| 198.92 | 0.0000 |
| 198.56 | 0.0054 |
| 197.78 | 0.0000 |
| 197.18 | 0.0006 |
| 196.96 | 0.0016 |
| 196.58 | 0.0017 |
| 195.85 | 0.0030 |
| 195.40 | 0.0106 |
| 195.38 | 0.0042 |
| 194.72 | 0.0058 |
| 193.83 | 0.0000 |
| 193.27 | 0.0177 |
| 192.42 | 0.0080 |
| 191.55 | 0.0000 |
| 191.08 | 0.0003 |
| 189.77 | 0.0000 |
| 189.27 | 0.0000 |
| 188.61 | 0.0000 |
| 187.49 | 0.0000 |
| 185.77 | 0.0038 |
| 184.01 | 0.0256 |
| 183.23 | 0.0115 |
| 181.58 | 0.0000 |
| 181.08 | 0.0001 |
| 180.63 | 0.0314 |

| | |
|---|---|
| 180.61 | 0.0163 |
| 179.21 | 0.0001 |
| 178.68 | 0.0032 |
| 175.72 | 0.0000 |
| 175.68 | 0.0054 |
| 173.95 | 0.0104 |
| 173.54 | 0.0050 |
| 173.31 | 0.0013 |
| 173.27 | 0.0017 |
| 172.24 | 0.0008 |
